# Transcriptomic differences in chromatin and cell cycle regulation in A549 cells after irradiation with carbon ions and X-rays

**DOI:** 10.3389/fonc.2026.1869974

**Published:** 2026-07-14

**Authors:** Hasan Nisar, Özdemirhan Serçin, Christine E. Hellweg

**Affiliations:** 1 Department of Radiation Biology, Institute of Aerospace Medicine, German Aerospace Center (DLR), Cologne, Germany; 2 Department of Medical Sciences, Pakistan Institute of Engineering and Applied Sciences (PIEAS), Islamabad, Pakistan

**Keywords:** A549 lung cancer, cell cycle regulation, chromatin regulatory reprogramming, DNA damage response, gene set enrichment analysis (GSEA), high-LET radiation, RNA sequencing

## Abstract

**Introduction:**

High-linear energy transfer (LET) radiation such as carbon ions exhibits greater biological effectiveness than conventional low-LET X-rays, but the transcriptional mechanisms underlying this advantage remain incompletely understood. We hypothesized that high-LET radiation induces a qualitatively different transcriptional response rather than simply amplifying low-LET signaling.

**Methods:**

A549 non-small cell lung cancer cells were exposed to equal physical doses (8 Gy) of X-rays or carbon ions (LET 73 keV/µm), and transcriptomic profiling was performed 4 h post-irradiation. Differential expression analysis was integrated with Hallmark pathway enrichment using gene set enrichment analysis (GSEA), over-representation analysis (ORA), and leading-edge gene interrogation to identify shared and LET-dependent gene expression regulation.

**Results:**

Both radiation modalities activated a conserved DNA damage response characterized by p53 signaling and apoptosis-related genes. In contrast, carbon ions selectively suppressed mitotic regulators including CENPE, KIF2C, PLK1, and BUB1, consistent with transcriptional disruption of the replication–segregation machinery. High-LET irradiation additionally enriched inflammatory and stress-associated pathways, including tumor necrosis factor (TNF), Nuclear Factor κB (NF-κB) and extracellular matrix and adhesion-related signatures annotated within the Hallmark epithelial–mesenchymal transition (EMT) gene set. Carbon ions also downregulated multiple core and linker histone genes, revealing a chromatin regulatory reprogramming signature although this may reflect modulation of mRNA stability linked to replication stress and cell-cycle progression. KRAS-associated gene networks were enriched under high-LET conditions, reflecting convergence of stress-responsive signaling.

**Discussion:**

At equal physical doses, high-LET carbon ion irradiation is associated with a transcriptional program distinct from that of low-LET X-rays, characterized by downregulation of mitotic and chromatin regulatory programs and selective engagement of stress-associated signaling networks. These findings provide mechanistic insight into LET-dependent radiobiology and suggest transcriptional pathway remodeling may contribute to the enhanced biological effectiveness of carbon ions.

## 1 Introduction

Lung cancer remains one of the leading causes of cancer-related mortality worldwide, and radiotherapy continues to play a central role in both curative and palliative treatment strategies (1). However, intrinsic and acquired radioresistance of tumor cells, together with dose-limiting toxicity to surrounding normal tissues, restrict therapeutic gain. These limitations have driven sustained interest in strategies to enhance tumor control while minimizing normal tissue injury. Such approaches include integration of systemic therapies such as immunotherapy (2), optimization of dose delivery through altered fractionation (3) and functional imaging–guided dose escalation (4, 5), and the use of high-linear energy transfer (LET) radiation (6).

High-LET radiation, such as carbon ions, differs fundamentally from conventional low-LET X-rays in both physical dose deposition and biological effects. Carbon ions produce densely ionizing tracks that generate complex DNA damage with clustered lesions occurring within one or two helical turns of DNA, which are more difficult to repair (7). These features are associated with enhanced relative biological effectiveness (RBE), increased clonogenic cell killing, altered checkpoint signaling, and elevated mitotic catastrophe (8, 9). Although these phenotypic advantages are well documented, the molecular programs and constituent genes that distinguish high-LET from low-LET radiation responses remain incompletely defined.

Several approaches have been employed to identify transcriptional signatures of high-LET radiation exposure. These include cross-modality comparisons of particle versus photon irradiation, dose–response transcriptomic profiling, and time-resolved expression analyses following heavy ion exposure (10–12). While such studies have reported pathway-level differences in DNA damage response, cell-cycle regulation, immune signaling, and metabolic reprogramming, there is limited consensus regarding high-LET gene expression signature. Reported profiles often vary substantially depending on radiation quality, dose, post-irradiation interval, and cellular background (13), making it difficult to distinguish generic radiation stress responses from true LET-dependent programs. Consequently, whether high-LET radiation induces a qualitatively distinct transcriptional state, rather than simply representing an amplified low-LET response remains an open question. In addition to transcriptional regulation, cellular responses to genotoxic stress are also shaped by post-transcriptional mechanisms, including regulation of mRNA stability and processing (14–16), which may contribute to early changes in transcript abundance independently of transcriptional activation.

In our previous work, carbon ion irradiation induced significantly greater clonogenic cell death, prolonged G2/M arrest and distinct cytokine secretion kinetics in A549 non-small cell lung cancer cells compared with X-ray exposure at the same physical dose (17–19). These findings suggested that high-LET radiation does not simply intensify DNA damage complexity but may drive a qualitatively distinct cellular stress state, consistent with its well-documented higher relative biological effectiveness (RBE) (20). The 2–6 h post-irradiation window is well established as the period of peak transcriptional induction of canonical DNA damage response (DDR) targets including CDKN1A, MDM2, DDB2, and FDXR. This makes 4 h an appropriate time point for capturing the primary radiation quality-specific transcriptional wave before secondary adaptive responses begin to dominate (21–23). Early post-irradiation transcript abundance may reflect not only transcriptional activation but also post-transcriptional regulation, including modulation of mRNA stability.

Here, we performed a head-to-head comparison of A549 cells exposed to equal physical doses (8 Gy) of low-LET X-rays or high-LET carbon ions (LET 73 keV/µm) with transcriptomic profiling at 4 h post-irradiation. We hypothesized that the greater RBE of carbon ions for cell killing may be underpinned by qualitatively distinct rather than simply quantitatively amplified transcriptional programs. To test this, we combined differential gene expression analysis with Hallmark pathway enrichment and leading-edge gene interrogation to determine whether high-LET irradiation preferentially enforces deeper cell-cycle suppression, perturbs chromatin regulatory programs, and engages distinct stress-response signaling networks compared with X-ray exposure.

The RNA-seq dataset used in this study was generated from A549 cells irradiated with X-rays or carbon ions. This dataset was the normoxia reference in our previously published studies examining hypoxia-related transcriptional responses in the same experimental system (17, 18, 24, 25).

## 2 Materials and methods

### 2.1 Cell culture

A549 human lung adenocarcinoma cells (male origin, KRAS-mutated, p53 wild-type (26) were obtained from LGC Genomics (Berlin, Germany). Cells were cultured in 25 cm² flasks (Labsolute, Th. Geyer GmbH, Renningen, Germany) at a seeding density of 5,000 cells/cm² in α-Minimal Essential Medium (α-MEM; PAN Biotech, Aidenbach, Germany) as described in our previous work (18). The culture medium was supplemented with 10% (v/v) dialyzed fetal bovine serum (FBS; PAN Biotech), 2% (v/v) sterile glucose solution (0.94 mol/L), 1% (v/v) penicillin (10,000 U/mL)/streptomycin (10 mg/mL), 1% (v/v) neomycin/bacitracin (Biochrom AG, Berlin, Germany), and 1% (v/v) amphotericin B (250 μg/mL; PAN Biotech).

Cells were routinely screened for mycoplasma contamination by polymerase chain reaction analysis of culture supernatants (Leibniz-Institut DSMZ, Braunschweig, Germany) and were confirmed to be mycoplasma-free.

Cultures were maintained at 37 °C under saturated humidity conditions (20% O_2_, 5% CO_2_; Heraeus HERAcell 150 incubator, Thermo Fisher Scientific, Karlsruhe, Germany) for 48 h prior to irradiation to ensure exponential growth.

### 2.2 Irradiation

Following 48 h of incubation, A549 cells were subjected to irradiation using either X-rays or carbon ions (¹²C).

X-ray irradiation (LET: 0.3–3.0 keV/μm) was carried out at the Institute of Aerospace Medicine (DLR, Germany) using an RS 225 X-ray chamber (X-strahl, Ratingen, Germany) at a dose rate of 1.0 Gy/min. This dose rate was maintained by positioning the samples at a fixed distance of 450 mm from the X-ray source. A 0.5 mm copper filter was employed to remove low-energy components of the beam. Cells were irradiated in culture flasks (25 cm²). Dose and dose rate were continuously monitored using a UNIDOS^webline^ dosimeter with a TM30013 ionization chamber (PTW, Freiburg, Germany).

Carbon ion irradiation was performed at the Grand Accélérateur National d’Ions Lourds (GANIL, Caen, France) at a dose rate of 1 Gy/min. Cells were positioned within the plateau region of the Bragg curve to ensure a uniform LET across the cellular layer. To achieve a clinically relevant LET in water (~73–75 keV/μm), the primary beam energy (95 MeV/n) was reduced to 35 MeV/n using a 16.9 mm polymethyl methacrylate (PMMA) energy degrader. Additional energy loss through the polystyrene base of the culture flask resulted in a final energy of 25.7 MeV/n and a calculated LET of 73 keV/μm. The corresponding residual range in water was approximately 2550 μm, confirming irradiation within the plateau region. Radiation dose for heavy ions was derived from particle fluence (particles/cm²). Due to the horizontal beam configuration during carbon ion exposure, culture flasks were maintained in an upright position and filled completely with medium to prevent desiccation.

Following irradiation, the medium was replaced and cells were returned to the incubator as described in our previous work (24).

### 2.3 Transcriptomic profiling by RNA sequencing

**Figure 1:**
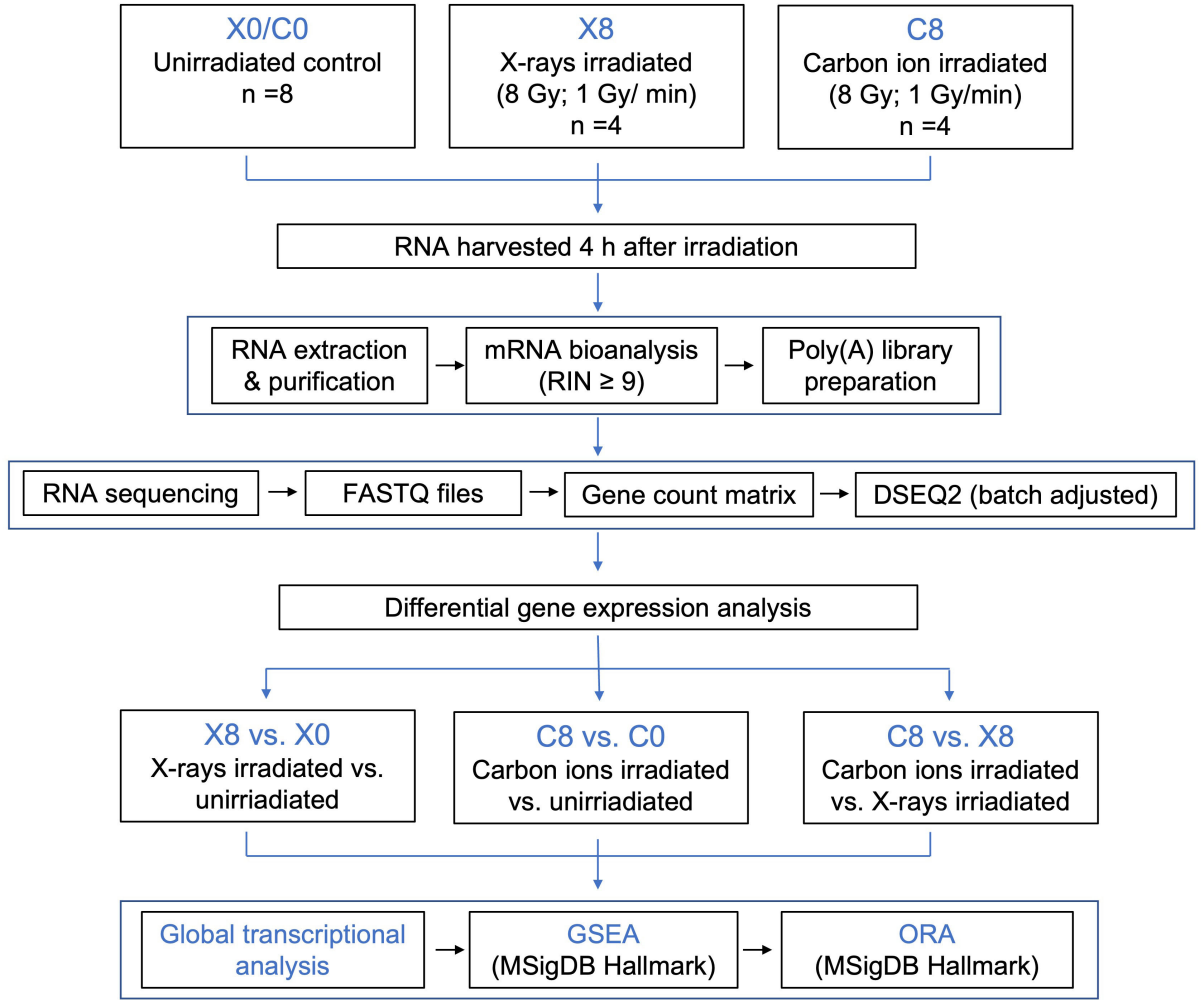
Study design and transcriptomic analysis pipeline for LET-dependent gene expression profiling. Cells were irradiated with X-rays (X8) or carbon ions (C8) at equal physical dose (8 Gy), alongside unirradiated controls (X0/C0). Three pairwise comparisons (X8 vs. X0, C8 vs. C0, C8 vs. X8) were analyzed using differential expression, followed by global transcriptomic assessment, GSEA, and ORA.

Global transcriptional profiling was performed in A549 cells maintained for 4 h at 37 °C and 5% CO_2_ following irradiation with 8 Gy of either X-rays (X8) or carbon ions (C8), or mock-irradiation (X0, C0) (**Figure 1**). At 4 h post-irradiation, culture media were removed and cells were lysed using RLT buffer (Qiagen, Hilden, Germany) supplemented with β-mercaptoethanol (1:100; Sigma-Aldrich, St. Louis, MO, USA).

Total RNA was isolated using the RNeasy Mini Kit (Qiagen) according to the manufacturer’s protocol. RNA quantity and integrity were assessed using the RNA 6000 Nano Assay on a Bioanalyzer system (Agilent Technologies, Böblingen, Germany). Only samples with an RNA Integrity Number (RIN) greater than 9.0 and a total yield of at least 3 μg RNA were included for downstream analysis (n = 4 independent biological replicates per condition, each derived from a separate cell passage).

RNA samples were transported on dry ice to GENEWIZ (Leipzig, Germany) for sequencing. mRNA libraries were prepared using poly(A) selection and sequenced on an Illumina NovaSeq 6000 platform (paired-end, 2 × 150 bp), generating approximately 350 million read pairs per run. Raw sequencing data were provided as FASTQ files by the sequencing facility.

Read alignment to the *Homo sapiens* reference genome (GRCh38) was performed by GENEWIZ using the STAR aligner. Gene-level counts were obtained from uniquely mapped reads within annotated exon regions.

### 2.4 RNA-seq data analysis

Downstream RNA-seq analysis was performed in R (version 4.5.2) software (27). The packages and versions used in the analysis are summarized in **Table 1**.

**Table 1:** Packages and versions used in RNA-seq data analysis.

Category	Tool/package	Version	Source	Access date
Environment	R	4.5.2	R Foundation for statistical computing	January 2026
Differential expression	DESeq2	1.50.2	Bioconductor	January 2026
Gene set enrichment (GSEA)	fgsea	1.36.2	Bioconductor	January 2026
Over-representation analysis (ORA)	clusterProfiler	4.18.4	Bioconductor	January 2026
Gene set database	msigdbr	25.1.1	CRAN	January 2026
Visualization	ggplot2	4.0.1	CRAN	January 2026
Heatmaps	pheatmap	1.0.13	CRAN	January 2026
Plot composition	cowplot	1.2.0	CRAN	January 2026
Data manipulation	dplyr	1.1.4	CRAN	January 2026
Data import	readr	2.1.6	CRAN	January 2026
String processing	stringr	1.6.0	CRAN	January 2026
Data structures	tibble	3.3.1	CRAN	January 2026
Data tidying	tidyr	1.3.2	CRAN	January 2026
Font rendering	showtext	0.9-7	CRAN	January 2026
Font handling	sysfonts	0.8.9	CRAN	January 2026

Raw integer gene counts derived from aligned reads were used for all analyses. Ensembl gene identifiers were used as primary keys; version suffixes were removed, and duplicate identifiers were collapsed by summation after suffix removal. Genes with at least 10 reads in at least two samples were retained.

Raw sequencing quality was assessed using FastQC for all 16 samples. Phred quality scores, where Q30 indicates 99.9% base call accuracy, were consistently high across all samples and conditions. Per-base scores were within the Q35–37 range across the full read length with no position-specific degradation, and greater than 93% of reads achieved Q30. GC content peaked consistently at approximately 50–52% across all samples, consistent with expected human transcriptome composition.

Differential expression analysis was performed using DESeq2 (version 1.50.2) with the design formula ~ batch + condition, where batch corresponded to irradiation date. Assessment of the design matrix confirmed no confounding between batch and condition. For exploratory quality assessment, variance-stabilizing transformation (VST; blind = FALSE, using fitted dispersion estimates from the design model) was applied, and principal component analysis (PCA) was performed to evaluate sample clustering. PCA confirmed clear separation of the three experimental conditions, with PC1 (51% variance) separating irradiated from unirradiated samples and further distinguishing carbon ion from X-ray conditions, and PC2 (27% variance) capturing residual biological variability. Batch was not a dominant source of variation. Full QC outputs including PCA plots and MA plots visualizing the differences between treatments, by transforming the data onto M (log2 fold change) and A (mean of normalized counted) scales, are provided as Supplementary Material (**Supplementary Figures A1**, **A2**). Normalization was performed using the median-of-ratios method implemented in DESeq2.

The following contrasts were evaluated:

- X8 vs. X0 (photon-mediated response)
- C8 vs. C0 (carbon-ion–mediated response)
- C8 vs. X8 (direct LET-dependent response)

Differential expression was assessed using Wald tests with Benjamini-Hochberg correction. Genes were considered significantly differentially expressed if they met both criteria: adjusted p-value < 0.05 and |log2 fold change| ≥ 1. No log2 fold-change shrinkage was applied. This threshold — equivalent to a minimum two-fold change — was applied to identify transcriptional changes of both statistical significance and biologically meaningful magnitude, consistent with the thresholded testing approach implemented in DESeq2 (lfcThreshold = 1, altHypothesis = “greaterAbs”), which tests the null hypothesis that the true fold-change lies within ±2-fold rather than at zero. This filtering strategy enriches for high-confidence, large-effect changes and should be interpreted in that context when evaluating pathway-level results.

Pathway-level analysis was performed using MSigDB Hallmark gene sets. Pre-ranked gene set enrichment analysis (GSEA) was conducted using fgsea (version 1.36.2). Genes were ranked using the DESeq2 Wald statistic. Over-representation analysis (ORA) was performed using clusterProfiler (version 4.18.4) on significantly differentially expressed genes, using the set of genes tested in differential expression analysis as background. Leading-edge genes and overlap analyses were used to identify shared and modality-specific transcriptional responses.

## 3 Results

### 3.1 Global transcriptional divergence between X-rays and carbon ions

To determine whether high-LET carbon ions elicit a transcriptional response that is qualitatively distinct from low-LET X-rays, we compared global gene expression profiles of A549 cells exposed to 8 Gy X-rays (X8) or 8 Gy carbon ions (C8) with unirradiated controls (X0 or C0), as well as directly between radiation qualities (C8 vs. X8).

**Figure 2:**
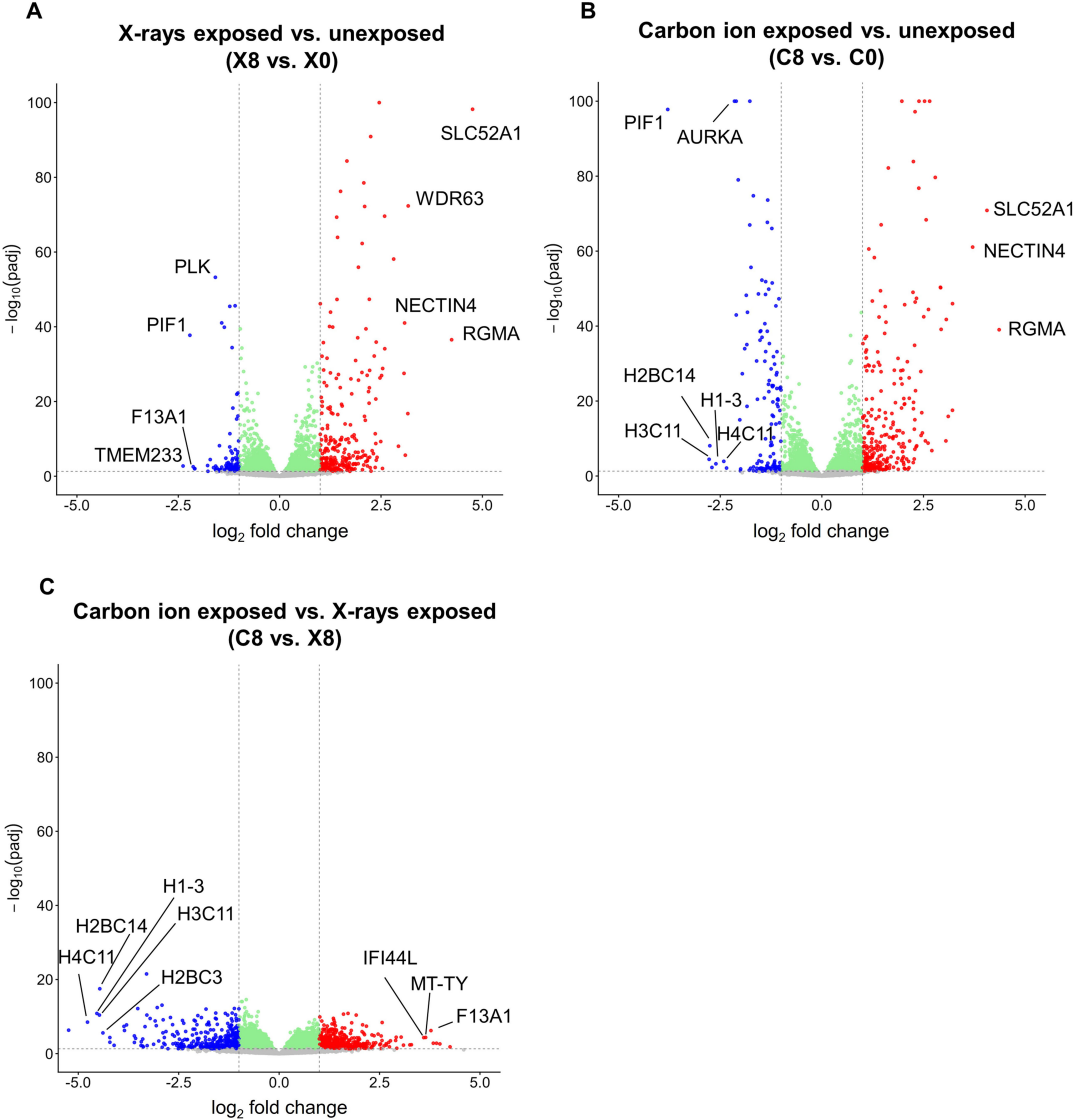
Differential gene expression following X-ray and carbon ion irradiation. Volcano plots depicting differential gene expression in A549 cells following **(A)** X-ray exposure (X8 vs. X0), **(B)** carbon ion exposure (C8 vs. C0), and **(C)** direct comparison of radiation qualities (C8 vs. X8). The x-axis shows the raw log2 fold change (log2FC) and the y-axis shows -log10 of the Benjamini-Hochberg adjusted p-value (padj). Vertical dashed lines indicate the |log2FC| = 1 threshold; horizontal dashed line indicates padj = 0.05. Genes are color-coded as follows: red, significantly upregulated (padj < 0.05 and log2FC > 1); blue, significantly downregulated (padj < 0.05 and log2FC < -1); green, statistically significant but below the fold-change threshold (padj < 0.05, |log2FC| ≤ 1); grey, not statistically significant. Significant genes with the highest log2FC that are of biological interest are labeled. Note the prominent downregulation of histone genes (H1-3, H2BC14, H3C11, H4C11) and replication-segregation regulators in panel C, reflecting the directional divergence between carbon ion and X-ray transcriptional responses at equal physical dose.

Volcano plots (**Figures 2A–C**) illustrate the global distribution of effect sizes and statistical significance across the three comparisons. Both radiation modalities induced widespread statistically significant (false discovery rate (FDR) < 0.05) transcriptional changes relative to unirradiated controls. X-ray exposure (X8 vs. X0) resulted in 318 upregulated and 111 downregulated (total 429) differentially expressed genes (DEGs), whereas carbon ion exposure (C8 vs. C0) yielded 298 upregulated and 142 downregulated (total 440) DEGs. In contrast, the direct comparison between carbon ions and X-rays (C8 vs. X8) identified a substantially larger set of significant DEGs, with 495 genes upregulated and 406 downregulated (total 901). This magnitude of divergence exceeds what would be expected from simple quantitative amplification of the low-LET response, and as shown in subsequent pathway-level analyses (Sections 3.3–3.5), the two modalities engage qualitatively distinct transcriptional programs.

**Figure 3:**
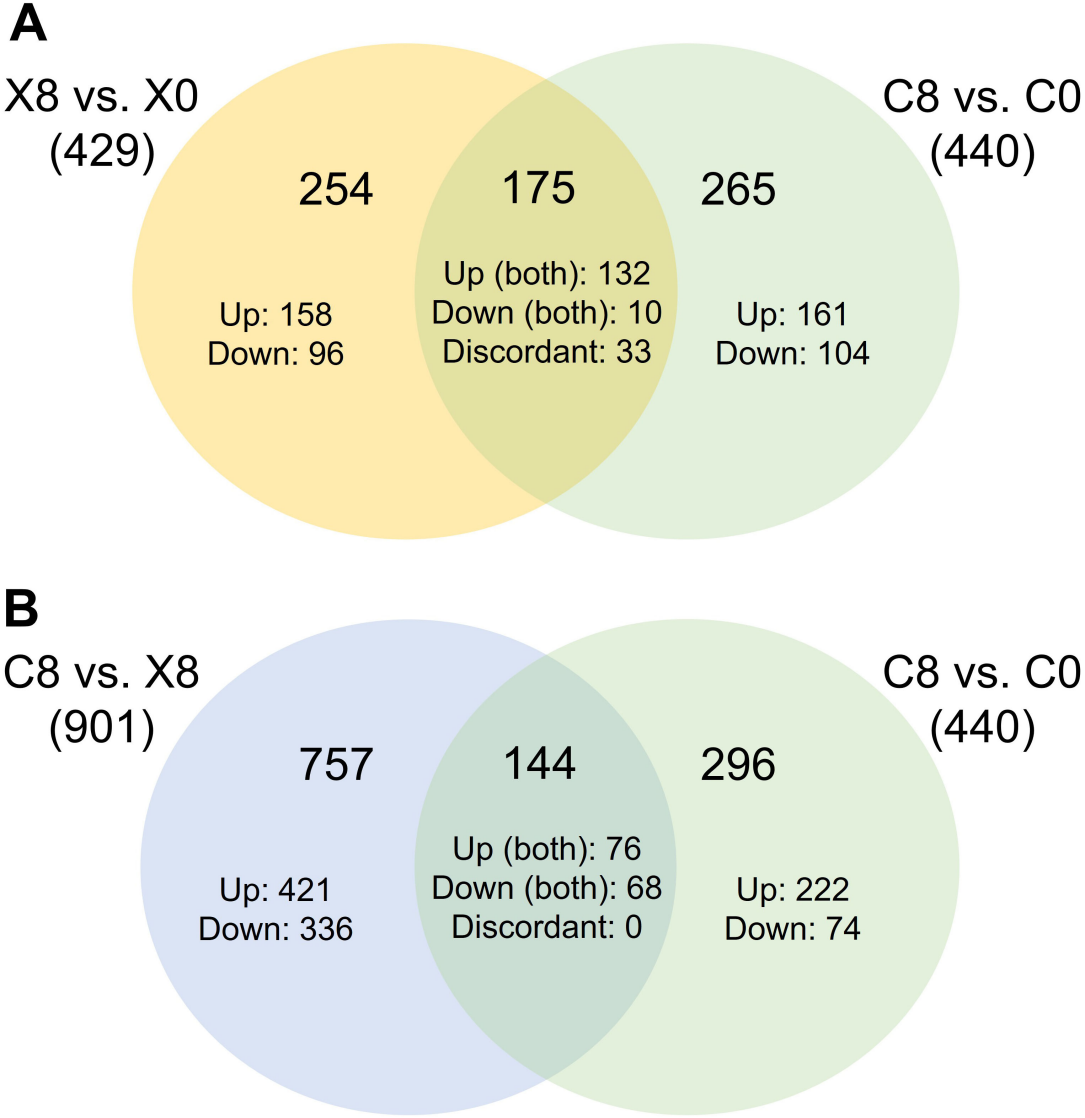
Overlap of significantly differentially expressed genes across irradiation contrasts. Venn diagrams illustrate shared and unique differentially expressed genes (FDR < 0.05, |log_2_FC| > 1) among comparison sets. **(A)** Overlap between X8 vs. X0 (429 genes) and C8 vs. C0 (440 genes). **(B)** Overlap between C8 vs. X8 (901 genes) and C8 vs. C0 (440 genes). In both panels, gene counts are further stratified by direction of regulation (up: upregulated, down: downregulated, and discordant: regulated in opposite directions between the two comparisons).

Of the 440 DEGs observed following carbon ion exposure relative to unirradiated controls (C8 vs. C0), 175 were also differentially regulated following X-ray exposure, as depicted in the Venn diagram (**Figure 3A**). However, 33 of these overlapping genes were regulated in opposite directions between the two radiation modalities, indicating discordant rather than shared regulation.

Of the 901 DEGs identified in the direct comparison (C8 vs. X8), 757 were not differentially regulated in carbon ion-exposed cells relative to unirradiated controls (C8 vs. C0), as shown in **Figure 3B**. This is a particularly important observation: it indicates that the majority of the transcriptional divergence between radiation qualities reflects genes actively modulated by X-rays that are not equivalently regulated by carbon ions, rather than genes uniquely driven by high-LET exposure alone. Together with the 33 discordantly regulated genes, this pattern is consistent with a genuinely distinct transcriptional program.

### 3.2 Histone gene expression downregulation following exposure to carbon ions vs. upregulation after X-rays exposure

A closer analysis of the 33 discordantly regulated genes revealed a striking enrichment of histone genes, indicating coordinated radiation quality-dependent regulation of chromatin-associated transcripts. Fifteen of the 33 discordant genes were histone genes spanning multiple families — linker histones (H1-3, H1-4, H1-5), canonical H2A clusters (H2AC), H2B clusters (H2BC), H3 variants (H3C), and H4 genes (H4C) — each significantly upregulated following X-ray exposure (X8 vs. X0; log_2_FC range +1.16 to +2.16) and simultaneously significantly downregulated following carbon ion exposure (C8 vs. C0; log_2_FC range −1.30 to −2.77), representing a clear bidirectional response across the full nucleosomal assembly program (**Table 2**).

**Table 2:** Histone genes regulated by carbon ion and X-rays exposure.

Significant genes	LFC*		
	X8 vs. X0	C8 vs. C0	C8 vs. X8
H1-3	2.12	-2.41	-4.54
H1-4	1.91	-1.30	-3.21
H1-5	1.37	-1.52	-2.89
H2AC12	1.82	-1.79	-3.60
H2AC17	1.79	-1.51	-3.30
H2AC21	1.94	-1.36	-3.29
H2BC13	1.59	-1.33	-2.92
H2BC14	1.71	-2.76	-4.46
H2BC17	1.81	-1.53	-3.34
H2BC18	1.93	-1.59	-3.52
H2BC6	1.16	-1.55	-2.71
H3C11	1.70	-2.77	-4.47
H3C13	1.21	-1.33	-2.54
H4C2	2.16	-2.61	-4.77
H4C5	1.54	-1.34	-2.89
H2AC13	1.15	-0.94	-2.09
H2AC14	1.56	-0.96	-2.52
H2AC16	1.14	-1.68	-2.82
H2AC4	1.67	-0.62	-2.29
H2BC3	1.67	-2.54	-4.21
H2BC10	2.40	-1.46	-3.86
H2BC15	1.28	-0.82	-2.10
H2BC21	1.06	-0.65	-1.71
H4C1	1.28	-0.76	-2.05
H4C13	2.17	-1.68	-3.85
H4C16	1.14	-0.43	-1.56
H4C3	1.11	-0.71	-1.82
H4C4	1.72	-0.89	-2.61
H4C8	1.17	-0.82	-2.00
H2AC11	0.29	-0.86	-1.15
H2AC20	0.75	-0.41	-1.16
H2BC11	0.46	-0.67	-1.12
H2BC4	0.83	-0.76	-1.58
H3C7	1.92	-0.72	-2.64

*Cells were irradiated with X-rays (X8) or carbon ions (C8) at equal physical dose (8 Gy), alongside unirradiated controls (X0/C0). Log_2_ fold change (LFC) of DEGs which do not meet the criteria of significance (p value < 0.05) and -1 < LFC > 1 are displayed in grey.

Direct comparison between radiation qualities (C8 vs. X8) revealed an additional 19 histone genes beyond the 15 formally discordant genes. Of these 19, fourteen were significantly upregulated following X-ray exposure (X8 vs. X0) but did not reach significance relative to unirradiated controls after carbon ion exposure (C8 vs. C0), while the remaining five showed only sub-threshold changes in both individual contrasts. Despite this gradient of effect sizes in the individual comparisons, all 19 reached significance in the direct C8 vs. X8 comparison, bringing the total number of histone genes demonstrating LET-dependent suppression to 34. This pattern indicates that the full extent of histone gene divergence between radiation qualities is not fully captured by comparing each modality to unirradiated controls alone, and is most sensitively revealed by direct head-to-head comparison. Notably, the magnitude of differential expression in the direct comparison consistently exceeded that observed in either individual contrast, reflecting additive divergence driven by simultaneous upregulation after X-rays and downregulation after carbon ions rather than suppression by carbon ions alone.

Together, these findings identify discordant histone gene regulation spanning all major nucleosomal histone families as a prominent and distinguishing feature of the transcriptional response to high-LET carbon ion irradiation, with the full extent of LET-dependent divergence most clearly captured in the direct C8 vs. X8 comparison.

### 3.3 Shared DNA damage response is dominated mainly by p53 signaling

To define the biological programs underlying the transcriptional differences between radiation qualities, we performed pre-ranked GSEA on all expressed genes for each comparison: X8 vs. X0, C8 vs. C0, and C8 vs. X8.

**Figure 4:**
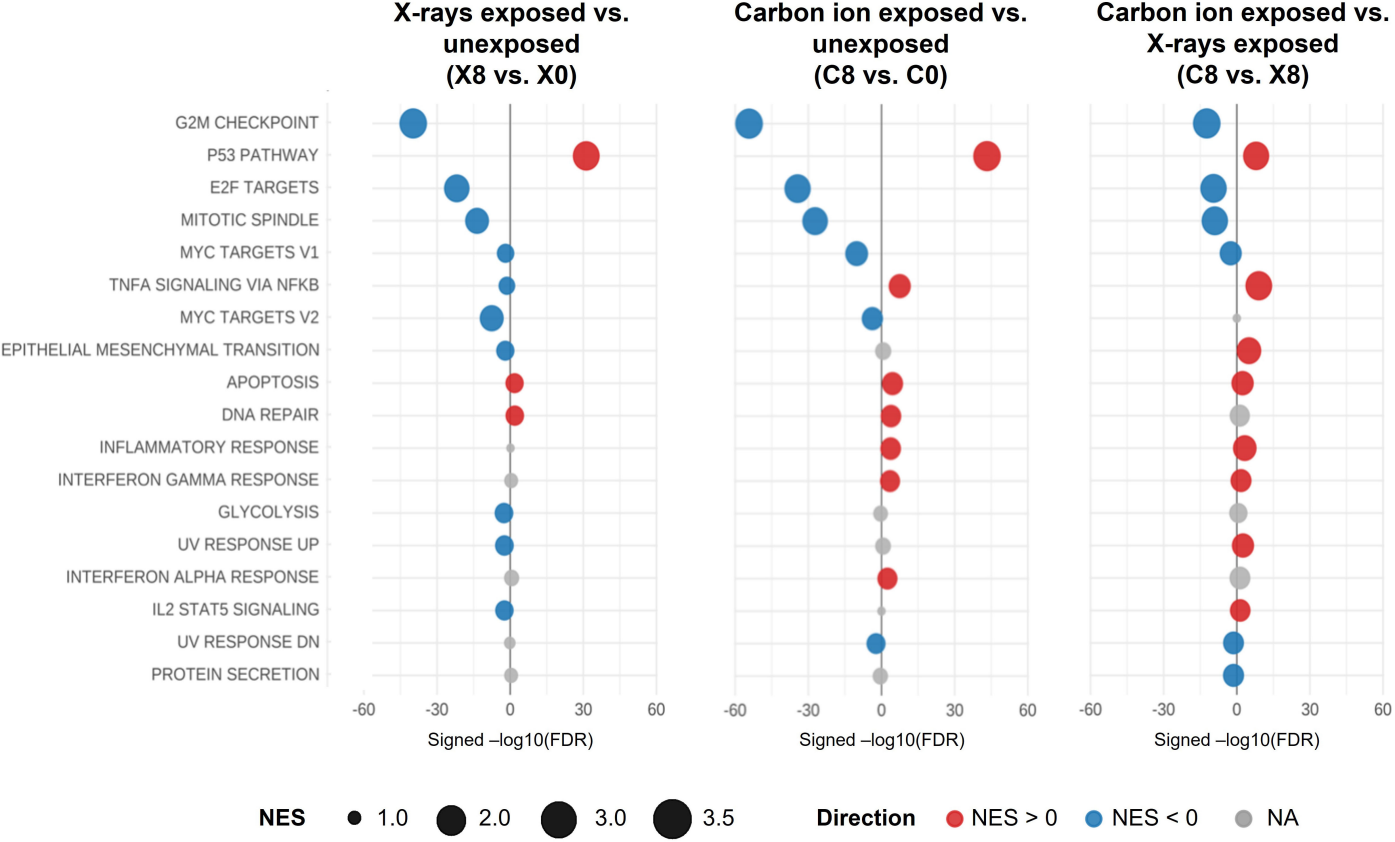
Comparative Hallmark pathway enrichment across irradiation contrasts. Dot plots showing pre-ranked GSEA results for three pairwise contrasts: X-ray-irradiated versus unirradiated controls (X8 vs. X0), carbon ion-irradiated versus unirradiated controls (C8 vs. C0), and carbon ion-irradiated versus X-ray-irradiated cells (C8 vs. X8). Each row represents one MSigDB Hallmark gene set. The x-axis shows signed -log10(FDR; Benjamini-Hochberg adjusted), where the sign is derived from the normalized enrichment score (NES): positive values (right of center line) indicate net upregulation of the gene set; negative values (left) indicate net downregulation. Dot color encodes direction: red, net upregulation (NES > 0); blue, net downregulation (NES < 0); grey, not significantly enriched (NA). Dot size reflects the absolute NES magnitude (see legend). Grey dots at or near zero indicate pathways that did not reach statistical significance in that contrast. Pathway names follow MSigDB Hallmark gene set nomenclature; for UV Response, UP and DN denote gene sets defined by upregulation and downregulation, respectively, in response to ultraviolet radiation; V1 and V2 denote two independently curated subsets of MYC target genes. Pathways are ordered consistently across all three panels to facilitate direct visual comparison. Note the selective enrichment of inflammatory and stress-associated pathways (TNF-α signaling via NF-κB, Epithelial Mesenchymal Transition) in the C8 vs. X8 comparison, and the deeper suppression of cell-cycle and replication-associated pathways (G2M Checkpoint, E2F Targets, Mitotic Spindle) relative to X8 vs. X0.

**Figure 5:**
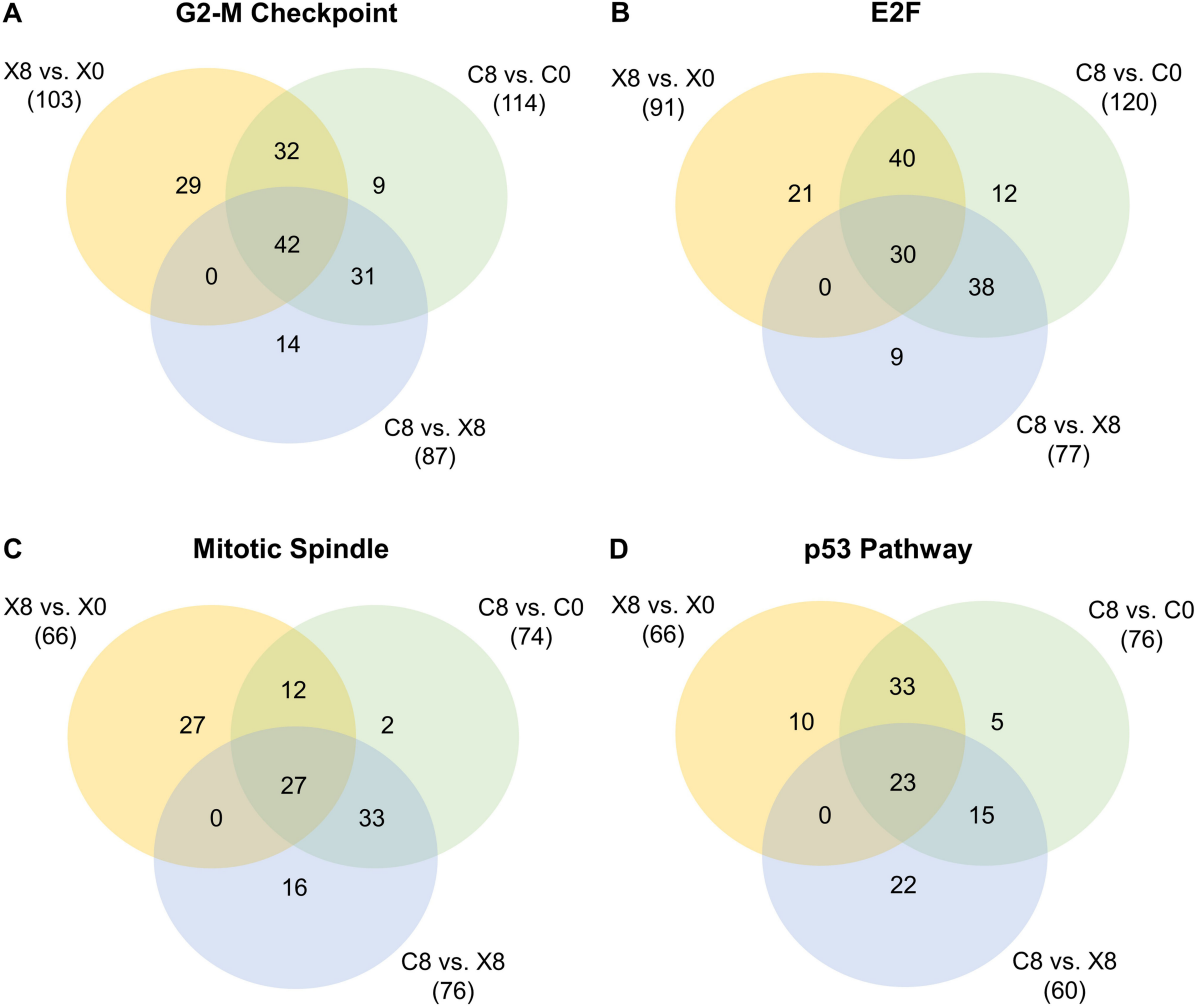
Leading-edge gene overlap within shared enriched pathways. Three-way Venn diagrams illustrating the overlap of leading-edge genes contributing to GSEA enrichment across three pairwise contrasts — X8 vs. X0, C8 vs. C0, and C8 vs. X8 — for four Hallmark gene sets: **(A)** G2M Checkpoint, **(B)** E2F Targets, **(C)** Mitotic Spindle, and **(D)** p53 Pathway. Numbers in each region indicate the count of leading-edge genes unique to or shared between contrasts. Total leading-edge gene counts per contrast are shown in parentheses beside each circle label. Note the substantial carbon ion-specific leading-edge gene subset in G2M Checkpoint, E2F Targets, and Mitotic Spindle panels — genes present in C8 vs. C0 and/or C8 vs. X8 but not X8 vs. X0 — reflecting deeper engagement of cell-cycle suppression programs following carbon ion irradiation. The p53 Pathway panel shows predominantly shared leading-edge genes across both radiation modalities, consistent with a conserved core DNA damage surveillance response. Complete gene lists for all regions are provided as **Supplementary Excel File**.

Across all three comparisons, enrichment was dominated by upregulation of the p53 pathway and downregulation of G2/M checkpoint, E2F targets, and mitotic spindle gene sets (**Figure 4**), indicating a conserved core radiation response shared between the two radiation qualities. Despite this shared core response, the overall enrichment profiles and specific leading-edge genes differed markedly between radiation qualities (**Figures 5A–D**; **Supplementary Excel Tables**), suggesting that the two modalities engage overlapping but non-identical transcriptional programs.

**Figure 6:**
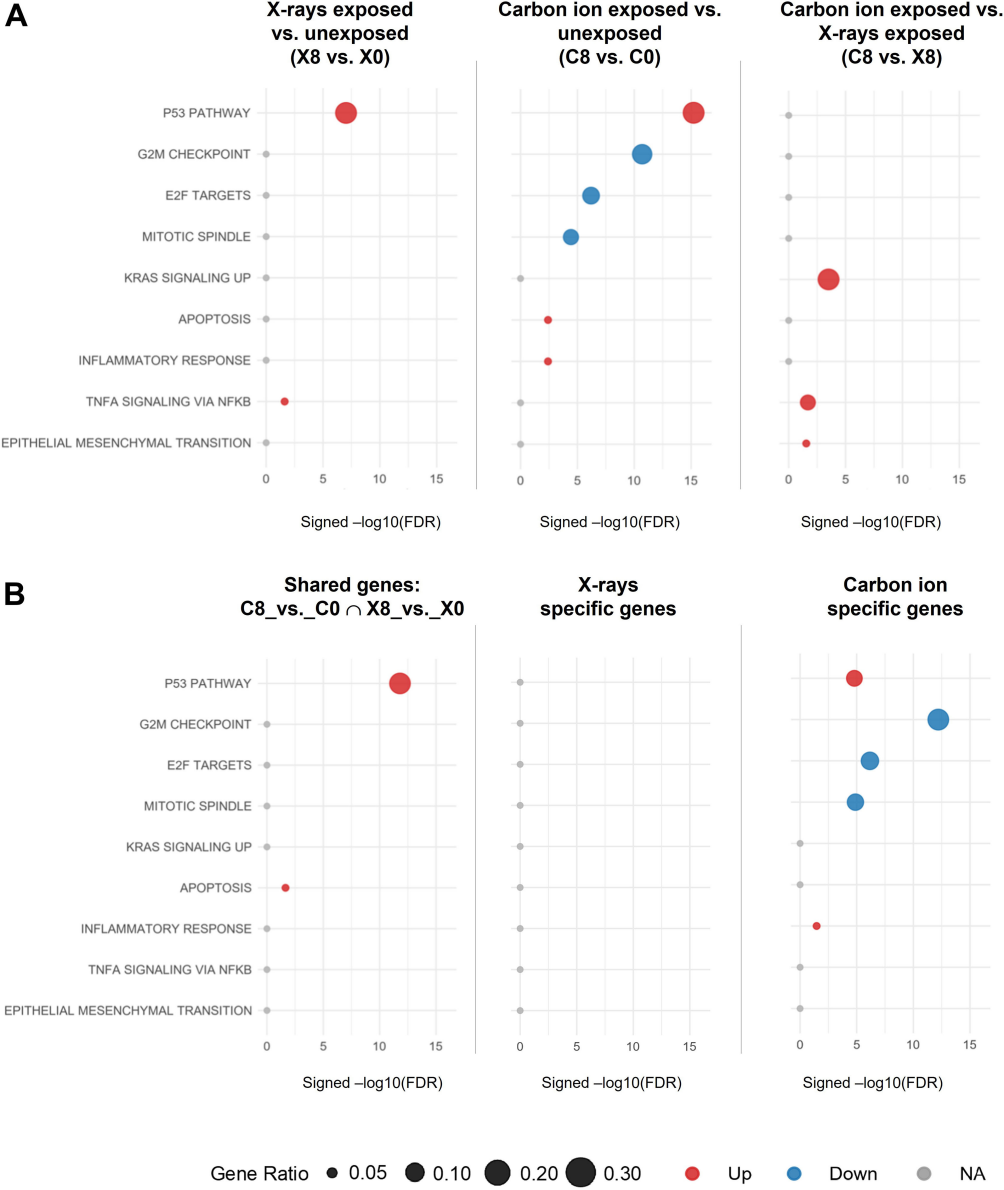
Over-representation analysis (ORA) of significant gene subsets across irradiation contrasts. Dot plots showing Hallmark pathway ORA results for significantly differentially expressed genes (padj < 0.05, |log2FC| ≥ 1). The x-axis shows -log10(FDR; Benjamini-Hochberg adjusted). Dot color indicates the predominant direction of regulation within each pathway: red, net upregulation; blue, net downregulation; grey, not significant (NA). Dot size reflects the gene ratio — the proportion of pathway genes present in the input gene set (see legend). **(A)** ORA performed separately on all DEGs from each pairwise contrast: X8 vs. X0, C8 vs. C0, and C8 vs. X8. **(B)** ORA stratified by modality specificity: genes shared between C8 vs. C0 and X8 vs. X0 (shared radiation response), X-ray-specific genes, and carbon ion-specific genes. Note the broader enrichment of cell-cycle suppression pathways — including G2M Checkpoint, E2F Targets, and Mitotic Spindle — in the carbon ion-specific subset relative to the X-ray-specific subset, and the conserved p53 pathway enrichment in the shared gene set.

ORA performed against the MSigDB Hallmark database identified the p53 pathway as the only gene set significantly enriched following exposure to either radiation quality relative to unirradiated controls (**Figure 6A**). In contrast, G2/M checkpoint, E2F targets, and mitotic spindle pathways were significantly enriched only following carbon ion exposure (C8 vs. C0), indicating that deeper repression of cell-cycle regulatory programs is a high-LET-specific feature not captured by X-ray exposure at the same physical dose.

### 3.4 High-LET carbon ions enforce deeper repression of mitotic replication–segregation programs

To further resolve the LET-dependent component of the transcriptional response, ORA was performed separately on three gene subsets stratified by overlap analysis: shared genes (significantly regulated in both C8 vs. C0 and X8 vs. X0), X-rays specific genes (significantly regulated in X8 vs. X0 only), and carbon ion specific genes (significantly regulated in C8 vs. C0 only) (**Figure 6B**). This stratified approach was adopted deliberately to isolate the pathway-level contributions of each gene subset, with the understanding that pre-stratification by direction of regulation will by design favor pathway enrichment within each subset. Results should therefore be interpreted in the context of this analytical framework rather than as independent pathway discovery.

ORA of shared genes confirmed p53 pathway as the primary concordant response to both radiation qualities. ORA of X-rays specific genes produced no significant pathway enrichment across any of the Hallmark gene sets examined, indicating that genes uniquely regulated by X-ray exposure do not cluster into coherent biological programs. In contrast, ORA of carbon ion specific genes demonstrated significant enrichment of G2M checkpoint, E2F targets, and mitotic spindle pathways — all downregulated — alongside additional enrichment of the p53 pathway and inflammatory response. The G2M checkpoint showed the strongest enrichment signal among all pathways and gene subsets examined. The appearance of an additional p53-associated gene subset specific to carbon ions, beyond the shared p53 response, indicates that high-LET irradiation engages a broader and more extensive p53-dependent transcriptional program than X-ray exposure at the same physical dose. The enrichment of inflammatory response among carbon ion specific genes further indicates that this program begins to emerge at the level of individual modality responses, extending into the secondary stress programs examined in Section 3.5.

This asymmetry between X-rays specific and carbon ion specific gene subsets, where only the latter organizes into coherent pathway-level programs, is consistent with carbon ion irradiation engaging a transcriptional program that differs in composition, and not only in magnitude, from that elicited by X-rays at the same physical dose. Individual significant DEGs contributing to the shared p53 response and to the LET-specific repression of the replication–segregation axis are presented in **Tables 3**–**5**.

**Table 3:** p53-related genes significantly regulated (|log2FC| ≥ 1) by both carbon ion and X-ray exposure compared to unirradiated controls.

p53-related genes	LFC*	
	C8 vs. C0	X8 vs. X0
FAS	2.24	2.21
CYFIP2TRAF4	1.24	1.40
DGKA	1.78	1.14
SESN1	2.25	2.10
CDKN1A	2.92	2.16
TNFSF9	2.01	1.06
DDB2	1.37	1.05
MDM2	2.39	2.25
XPC	1.46	1.43
BTG2	2.65	2.04
FDXR	2.10	1.94
ATF3	2.28	1.05
ANKRA2	1.28	1.25
POLH	1.64	1.66
PPM1D	1.97	1.50
TRIAP1	1.10	1.25
ZMAT3	1.46	1.27

*Log_2_ fold change (LFC) of the expression of p53-related genes after exposure to X-rays (X8) or carbon ions (C8) at equal physical dose (8 Gy), compared to unirradiated controls (X0/C0).

**Table 4:** Cell cycle related genes significantly regulated by only carbon ion exposure (|log2FC| ≥ 1), but not by X-rays exposure, relative to unirradiated controls.

G2-M Checkpoint			E2F targets			Mitotic spindle		
Genes	LFC*		Genes	LFC*		Genes	LFC*	
	C ions C8 vs. C0	X-rays X8 vs. X0		C ions C8 vs. C0	X-rays X8 vs. X0		C ions C8 vs. C0	X-rays X8 vs. X0
DBF4	-1.31	-0.52	DEPDC1	-1.83	-0.93	NDC80	-1.38	-0.63
HMMR	-1.90	-0.88	HMMR	-1.90	-0.88	TPX2	-1.90	-0.72
NDC80	-1.38	-0.63	LMNB1	-1.01	-0.64	TTK	-1.38	-0.52
TPX2	-1.19	-0.72	CDC20	-1.19	-0.87	LMNB1	-1.19	-0.64
TTK	-1.30	-0.52	CKS2	-1.08	-0.62	NEK2	-1.30	-0.89
LMNB1	-1.01	-0.64	DLGAP5	-1.20	-0.65	CENPF	-1.01	-0.54
CDC20	-1.19	-0.87	TOP2A	-1.03	-0.32	DLGAP5	-1.20	-0.65
NEK2	-1.13	-0.89	CDCA8	-1.74	-0.87	TOP2A	-1.03	-0.32
CENPF	-1.60	-0.54	ESPL1	-1.06	-0.58	ESPL1	-1.06	-0.58
CKS2	-1.08	-0.62	CENPE	-2.11	-0.83	KIF23	-1.22	-0.81
TOP2A	-1.03	-0.32	KIF2C	-1.33	-0.97	KIF11	-1.16	-0.52
TROAP	-1.11	-0.73	MKI67	-1.29	-0.49	CENPE	-2.11	-0.83
ESPL1	-1.06	-0.58	BUB1B	-1.10	-0.55	KIF2C	-1.33	-0.97
KIF23	-1.22	-0.81	RACGAP1	-1.39	-0.78	INCENP	-1.23	-0.68
KNL1	-1.13	-0.34	HMGB2	-1.42	-0.37	RACGAP1	-1.39	-0.78
KIF11	-1.16	-0.52	WEE1	-1.12	-0.59	BUB1	-1.21	-0.63
CENPE	-2.11	-0.83	KIF18B	-1.08	-0.68			
KIF2C	-1.33	-0.97	MXD3	-1.01	-0.88			
CCNA2	-1.38	-0.65						
MKI67	-1.29	-0.49						
INCENP	-1.23	-0.68						
RACGAP1	-1.39	-0.78						
CCNF	-1.77	-0.95						
BUB1	-1.21	-0.63						
UBE2C	-1.25	-0.69						

*Log_2_ fold change (LFC) of DEGs in cells that were irradiated with X-rays (X8) or carbon ions (C8) at equal physical dose (8 Gy), compared to unirradiated controls (X0/C0). LFC of DEGs which do not meet the criteria of significance (p value < 0.05) and -1 < LFC > 1 are displayed in grey.

**Table 5:** p53 and inflammation-related genes significantly regulated only by carbon ion exposure (|log2FC| ≥ 1), but not by X-rays exposure, relative to unirradiated controls.

p53 pathway			Inflammatory response		
Genes	LFC*		Genes	LFC*	
	C ions C8 vs. C0	X-rays X8 vs. X0		C ions C8 vs. C0	X-rays X8 vs. X0
TP63	-1.47	0.36	GNA15	1.84	1.10
TRAF4	1.55	0.72	EBI3	1.06	0.37
RNF19B	1.39	0.56	CCL20	-1.75	0.65
GADD45A	1.56	0.24	KCNJ2	1.46	0.62
NINJ1	1.01	0.84	CD70	1.05	0.63
ISCU	1.05	0.69	CCR7	1.11	0.51
RALGDS	1.07	0.65	PTAFR	1.00	0.91
RRAD	2.44	0.34	CHST2	1.25	-0.30
PLK3	1.81	0.52			
PHLDA3	1.87	0.96			
EPS8L2	1.41	0.92			
AEN	1.10	0.62			
RAP2B	1.16	0.81			
PLXNB2	1.01	0.47			
PDGFA	1.25	0.57			

*Log_2_ fold change (LFC) of DEGs in cells that were irradiated with X-rays (X8) or carbon ions (C8) at equal physical dose (8 Gy), compared to unirradiated controls (X0/C0). All LFC marked in yellow have to be displayed in grey, as these are not significant. Significant and non-significant regulation must be clearly distinguished.

### 3.5 LET-dependent secondary stress programs: TNF–NF-κB, EMT, and KRAS signatures

GSEA of the direct comparison (C8 vs. X8) identified selective enrichment of inflammatory and stress-associated pathways following carbon ion irradiation, most notably TNF-α signaling via NF-κB and the epithelial–mesenchymal transition (EMT) gene set (**Figure 4**). Neither pathway was enriched with either radiation quality relative to unirradiated controls, indicating that these programs reflect LET-dependent transcriptional divergence rather than a general radiation response. ORA of the C8 vs. X8 comparison corroborated these findings and additionally identified significant enrichment of KRAS-associated gene networks (**Figure 6A**). Significant DEGs contributing to TNF–NF-κB, EMT, and KRAS pathway enrichment are listed in **Table 6**.

**Table 6:** Genes significantly regulated (|log2FC| ≥ 1) by carbon ion exposure relative to X-rays-irradiated cells.

KRAS signaling		TNFα signaling via NFκB		Epithelial mesenchymal transition (EMT)	
Genes	LFC* C8 vs. X8	Genes	LFC* C8 vs. X8	Genes	LFC* C8 vs. X8
USH1C	-2.38	TNC	1.07	TNC	1.07
TNFRSF1B	1.25	HBEGF	1.15	COL11A1	-1.31
HBEGF	1.15	CCL20	-2.40	MYL9	-1.68
CCL20	-2.40	GADD45A	1.32	LAMA1	1.51
CXCR4	1.81	KLF9	-1.28	COL7A1	1.03
INHBA	-1.78	EGR1	1.57	GADD45A	1.32
F13A1	3.78	INHBA	-1.78	INHBA	-1.78
GADD45G	1.24	FOSB	2.57	SERPINE2	1.38
ANO1	1.09	KLF4	1.35	ECM1	1.20
EMP1	1.38	ATF3	1.23	PTX3	1.36
KLF4	1.35	PTX3	1.36	EDIL3	2.17
PTPRR	2.08	CSF2	1.37	ANPEP	1.22
ITGB2	1.09	IL7R	1.40	SNTB1	1.38
CSF2	1.37	PHLDA2	1.36	FOXC2	1.23
IL7R	1.40	MAFF	1.27		
TMEM158	1.44				
H2BC3	-4.21				

*Log_2_ fold change (LFC) of DEGs in cells that were irradiated at equal physical dose (8 Gy) with carbon ions (C8) compared to X-rays-exposed cells (X8).

Together, these findings demonstrate that radiation quality not only influences the magnitude of transcriptional change but also reshapes the composition of pathway-level stress responses, with high-LET irradiation selectively engaging inflammatory and stress-associated programs not observed following low-LET exposure at the same physical dose.

## 4 Discussion

This study identifies coordinated disruption of chromatin assembly and replication-associated transcriptional programs as a prominent feature of early response (4 h post-irradiation) to high-LET carbon ion irradiation in A549 lung cancer cells. In a head-to-head comparison using equal physical doses, both X-ray and carbon ion exposure activated a conserved DNA damage response characterized by p53-associated transcription. However, carbon ion irradiation was associated with a distinct transcriptional profile characterized by deeper repression of replication and mitotic networks, opposing regulation of histone gene expression, and selective activation of inflammatory and mesenchymal stress pathways. These transcriptional patterns align with our previously reported functional phenotypes, including enhanced clonogenic cell killing, prolonged G2/M arrest, and altered cytokine secretion following carbon ion exposure (17–19, 24). Together, these findings support the concept that high-LET radiation engages transcriptional programs that differ not merely in magnitude but also in regulatory composition.

### 4.1 Shared core radiation response is p53-mediated

Both X-ray and carbon ion irradiation strongly upregulated the p53 pathway along with a more subtle upregulation of apoptosis and DNA repair as revealed by GSEA (**Figure 4**). 56 leading edge genes of the p53 pathway were concordant between the two radiation qualities (**Figure 5D**; **Supplementary Excel Tables**), and ORA identified 17 significant DEGs from the p53 pathway co-upregulated after X-rays and carbon ions exposure (**Table 3**). This coordinated gene set reflects the canonical early genome surveillance response to DNA damage, in which radiation-induced activation of ATM/ATR kinases stabilizes p53, enabling transcriptional regulation of genes involved in cell-cycle checkpoint enforcement (CDKN1A, BTG2, ZMAT3), nucleotide excision and trans-lesion repair (DDB2, XPC, POLH), oxidative stress modulation (SESN1, FDXR), and apoptotic priming (FAS, TNFSF9, TRIAP1, ATF3), integrating arrest, repair facilitation, and apoptosis to prevent propagation of damaged cells (28, 29).

ORA additionally revealed that carbon ion exposure significantly modulated a further subset of p53-associated genes beyond the shared response (**Table 4**). Several of these are linked to reinforcement of checkpoint and stress-response signaling: GADD45A represents a canonical DNA damage–responsive mediator within the p53 stress network (30), whereas PHLDA3, a direct p53 target, antagonizes AKT signaling and promotes growth suppression under stress conditions (31), and AEN, a p53-inducible nuclease, participates in p53-dependent apoptotic execution (32). Induction of RRAD and ISCU, both linked to p53-mediated metabolic regulation and mitochondrial homeostasis, suggests engagement of broader metabolic stress pathways in response to high-LET damage (33, 34). Carbon ions additionally regulated several signaling-associated genes including TRAF4, RALGDS, RAP2B, PLXNB2, and PDGFA, which may reflect integration of p53 signaling with cytoskeletal stress adaptation (35–38). Conversely, TP63 was downregulated, potentially indicating suppression of p53-family programs linked to epithelial identity, regeneration and proliferation (39, 40). Together, this extended p53-associated gene set indicates that high-LET irradiation engages a broader and more extensive p53-dependent transcriptional program than X-ray exposure at the same physical dose, consistent with the more severe and complex genomic injury induced by carbon ions.

### 4.2 Replication–chromatin axis downregulation after exposure to high-LET radiation

High-LET irradiation induces a coordinated transcriptional state that simultaneously suppresses mitotic progression and alters chromatin regulatory programs, indicating disruption of a unified replication–chromatin axis. To resolve the structure of this response, we separately discuss its impact on the replication–segregation machinery and histone gene expression.

#### 4.2.1 Downregulation of the replication–segregation machinery following high-LET irradiation

While GSEA indicated that both radiation qualities downregulated G2/M checkpoint, E2F targets, and mitotic spindle pathways (**Figure 4**), with 39–74 concordant leading-edge genes shared across these pathways (**Figures 5A–C**), ORA of carbon ion specific genes demonstrated selective downregulation of a substantial subset of high-confidence genes within these pathways (25 G2/M-related, 18 E2F-associated, and 16 mitotic spindle genes; **Table 4**), with no equivalent pathway enrichment observed among X-rays specific genes.

These three Hallmark categories converge on a shared functional axis governing DNA replication competence, spindle assembly, kinetochore attachment, and chromosome segregation. The extensive gene-level overlap between pathways indicates that high-LET radiation exposure enforces coordinated transcriptional suppression of the entire mitotic program. Key downregulated genes included core spindle and kinetochore components such as CENPE, CENPF, NDC80, KNL1, TPX2, KIF11, KIF2C, and INCENP (41–43), as well as central mitotic checkpoint regulators including CDC20, BUB1, CCNF, TTK, RACGAP1, and ESPL1 (43, 44). Coordinated repression of these genes is consistent with inhibition of the machinery required for faithful mitotic progression rather than simple slowing of proliferation, consistent with the known ability of high-LET radiation to generate densely clustered and complex DNA lesions that prolong checkpoint activation and increase the likelihood of mitotic failure (10, 45). This transcriptional pattern is consistent with sustained enforcement of a G2/M blockade and impaired restoration of replication–segregation competence at 4 h post irradiation. It aligns with the prolonged G2/M arrest and reduced clonogenic survival previously observed in this model (17–19).

#### 4.2.2 Discordant histone gene expression reveals LET-dependent chromatin regulatory reprogramming

A prominent feature of the carbon ion response was coordinated discordant regulation of histone gene expression, observed concurrently with repression of mitotic regulators (**Table 2**). This pattern did not affect isolated histone variants; rather, it spanned linker histones (H1), canonical H2A and H2B clusters, H3 variants, and H4 genes, indicating broader modulation of nucleosomal assembly programs. X-ray exposure produced consistent upregulation of these genes at 4 h post-irradiation (log_2_FC approximately +1.1 to +2.4). Carbon ion irradiation, by contrast, resulted in their pronounced downregulation (approximately −1.3 to −2.8). The direct comparison (C8 vs. X8) revealed the magnitude of this divergence. Log_2_FC values ranged from −1.12 to −4.77, reflecting additive separation driven by simultaneous upregulation after X-rays and downregulation after carbon ions.

This bidirectional response indicates radiation quality-specific control of chromatin regulatory programs rather than nonspecific suppression secondary to reduced proliferation. Both radiation qualities downregulated mitotic and replication-associated genes. However, histone transcription displayed opposing transcriptional responses.

Histone gene expression is tightly integrated with DNA damage signaling and cell-cycle checkpoint control. Histone transcripts are among the most rapidly repressed following irradiation (46). This repression is driven by checkpoint-dependent inhibition of cyclin E-Cdk2 activity, which in turn suppresses the histone transcriptional activator NPAT (47). Expression recovers as cells progress toward repair and chromatin restoration. Bulk transcriptional recovery has been shown to begin as early as 2 h post-irradiation, coinciding with reduction of the DNA damage signal (46). The modest upregulation of histone transcripts in X-ray-exposed cells at 4 h is consistent with this recovery trajectory. By 4 h, γH2AX burden was substantially reduced in X-ray-exposed cells relative to 1 h post-irradiation (17, 18), consistent with active repair progression and partial checkpoint resolution. Carbon ion-exposed cells, which sustain more complex and persistent lesions, remain in a deeper checkpoint-arrested state at the same time point, consistent with continued histone transcript suppression. The directional difference between radiation modalities at 4 h may therefore reflect different positions along the temporal trajectory of histone regulation.

In addition to transcriptional suppression, the early 4 h time point raises the possibility that post-transcriptional mechanisms contribute to the observed changes in histone transcript abundance. Unlike polyadenylated mRNAs, histone transcripts are stabilized through a conserved 3′ stem-loop structure recognized by the stem-loop binding protein SLBP (48). Histone mRNA stability is therefore tightly coupled to S-phase progression and replication fork activity, and is disrupted under conditions of replication stress or checkpoint enforcement (49). We propose that the sustained suppression of replication-segregation machinery observed following carbon ion irradiation, including downregulation of CENPE, KIF2C, BUB1, PLK1, CDC20, and TTK, may deprive SLBP of its replication-coupled stabilization signal, potentially amplifying histone mRNA destabilization independently of transcriptional repression. This model remains speculative and would require direct measurement of SLBP protein levels, histone transcript stability, and replication fork dynamics for validation. The impact of high-dose low- and high-LET ionizing radiation on histone mRNA stability through this mechanism remains to be examined.

### 4.3 LET-dependent secondary stress programs

In addition to core disruption of the replication–chromatin axis, high-LET irradiation induces a secondary layer of transcriptional responses reflecting cellular stress adaptation. These programs encompass inflammatory signaling, structural remodeling, and stress-network integration, and were examined through analysis of TNF–NF-κB signaling, EMT-associated transcription, and KRAS-related gene networks.

#### 4.3.1 High-LET irradiation induces TNF–NF-κB associated stress signaling

GSEA identified enrichment of stress and cytokine-associated transcriptional programs following carbon ion irradiation, most prominently TNF-α signaling via NF-κB, together with interferon-α and interferon-γ–related pathways (**Figure 4**). ORA further demonstrated regulation of 15 significant genes within the TNF-α–NF-κB signature (**Table 6**), with most genes showing increased expression alongside a smaller subset of downregulated genes. This mixed directionality suggests selective remodeling of TNF–NF-κB–associated networks rather than uniform activation, consistent with a stress-adaptive transcriptional response rather than a canonical inflammatory program.

Importantly, this signature was largely composed of immediate-early stress response regulators (EGR1, FOSB, ATF3) (50–52), cytokine-associated signaling mediators (HBEGF, CSF2, IL7R, PTX3) (53, 54), and DNA damage–linked genes such as GADD45A (55), rather than classical immune effector molecules. Together, these findings indicate convergence between DNA damage signaling, stress-response transcription factors, and NF-κB–associated gene networks following high-LET irradiation and the associated replication–chromatin stress state. Although TNF–NF-κB signaling is frequently associated with immune regulation in *in vivo* systems (56), this inflammatory-like transcriptional program emerged in a tumor cell–only *in vitro* system lacking immune or stromal components. Therefore, it can be interpreted as a model of cell-intrinsic stress signaling triggered by severe genomic injury. To evaluate a potential immune modulation or immune evasion, an experimental model including immune cells would be required. Whether similar signatures subsequently shape immune interactions in more complex microenvironmental contexts remains an important question for future investigation.

#### 4.3.2 Stress-associated extracellular matrix remodeling signature following carbon ion irradiation

GSEA demonstrated relative enrichment of the Hallmark EMT gene set in carbon ion-irradiated cells compared with X-ray-exposed cells (C8 vs. X8), whereas X-ray exposure alone (X8 vs. X0) produced modest suppression of this signature (**Figure 4**). The suppression of EMT-associated genes following X-ray exposure is consistent with reports of radiation-induced epithelial stabilization at early time points prior to the onset of later adaptive remodeling (57), and contrasts with the structural stress response engaged by carbon ions. ORA confirmed differential regulation of genes within the Hallmark EMT set in the C8 vs. X8 comparison, with 11 genes upregulated and 3 downregulated (**Table 6**). The upregulated genes were predominantly extracellular matrix (ECM) and adhesion-associated (TNC, LAMA1, COL7A1, SERPINE2, ECM1, EDIL3) (58, 59), whereas INHBA, MYL9, and COL11A1 — genes more commonly linked to mesenchymal programs — were downregulated. Notably, classical EMT master regulators including SNAI1/2, ZEB1/2, and TWIST1 were not among the significantly differentially regulated genes, and the absence of coordinated epithelial-to-mesenchymal marker switching further argues against canonical EMT activation.

This mixed directionality — with upregulation of ECM and adhesion genes and concurrent downregulation of mesenchymal-associated genes, in the absence of canonical EMT transcription factor induction — indicates that the observed enrichment does not represent coordinated induction of a classical invasive or metastatic program. Rather, it reflects selective extracellular matrix and adhesion-related transcriptional remodeling in the context of severe genomic stress and sustained checkpoint enforcement (57). Enrichment of the Hallmark EMT gene set following carbon ion irradiation is therefore more consistent with stress-induced cytoskeletal and ECM adaptation as part of a broader damage-response network than with acquisition of mesenchymal identity and should not be interpreted as evidence of EMT activation in the classical sense.

#### 4.3.3 KRAS signaling reflects stress-network convergence after exposure to high-LET radiation

Although not highlighted by GSEA, ORA identified significant regulation of KRAS-associated genes in the C8 vs. X8 comparison, including 13 upregulated genes (log_2_FC 1.15 to 3.78) and 4 downregulated genes (log_2_FC −1.78 to −4.21) (**Table 6**). This enrichment should not be interpreted as oncogenic KRAS activation or induction of a proliferative program. Rather, KRAS signaling is known to integrate extracellular and intracellular stress cues and regulate transcriptional programs involved in inflammatory signaling and adaptive responses to injury, independent of oncogenic transformation (60). Several of the identified DEGs — including TNFRSF1B, CSF2, CCL20, IL7R, HBEGF, KLF4, and CXCR4 — are downstream targets of MAPK and/or NF-κB (61, 62), activated in response to cellular stress and DNA damage. F13A1, the most strongly upregulated KRAS pathway gene, is involved in stress-related cytoskeletal remodeling (63), whereas H2BC3, the most strongly downregulated, is a core H2B histone already discussed in Section 4.2.2 and is only indirectly linked to the KRAS pathway through gene set annotation.

KRAS pathway enrichment therefore most likely reflects convergence of stress-responsive signaling networks activated by severe high-LET radiation-induced genomic damage, supported by the concurrent suppression of mitotic regulators, histone genes, and cell-cycle progression observed in the same samples. This indicates a dominant transcriptional state characterized by checkpoint enforcement, chromatin regulatory reprogramming, and proliferative arrest rather than growth promotion. Collectively, KRAS-associated enrichment represents engagement of shared stress-response circuitry within the broader high-LET radiation-induced transcriptional state, alongside sustained checkpoint activation, chromatin regulation, and inflammatory signaling.

It should be noted that A549 cells carry a constitutively activating KRAS G12S mutation, which results in elevated baseline RAS-MAPK signaling and may influence the expression of KRAS-associated downstream targets prior to irradiation. The KRAS-associated enrichment we observe following carbon ion irradiation therefore occurs against a background of already-elevated KRAS pathway activity, and it is possible that the KRAS-mutant context modifies A549 cells’ stress-induced engagement of MAPK and NF-κB downstream targets. Whether similar KRAS-associated enrichment would be observed in KRAS wild-type NSCLC cells following carbon ion irradiation cannot be determined from the present data and represents an important question for future investigation in additional cellular models.

### 4.4 Implications for the therapeutic advantage of high-LET radiation

The transcriptional programs characterized in this study provide contextual insight into the superior biological effectiveness of carbon ions previously documented in functional assays using the same model system (17). Carbon ion irradiation at equal physical doses was associated with a coordinated early transcriptional state at 4 h post-irradiation, characterized by deeper checkpoint enforcement, replication-segregation downregulation, chromatin regulatory suppression, and convergence of inflammatory and stress-associated signaling networks. These features were not observed to the same extent following X-ray exposure under identical conditions.

This early transcriptional profile is consistent with the prolonged G2/M arrest and reduced clonogenic survival previously reported in this model (17–19), though whether the transcriptional state observed at 4 h directly underlies these longer-term functional outcomes cannot be established without time-course data. Nonetheless, these findings identify the replication-chromatin axis as a candidate regulatory program warranting further investigation in the context of particle therapy.

For example, carbon ion irradiation was associated with pronounced suppression of mitotic regulators including PLK1 and BUB1, targets for which clinical inhibitors exist. Combining carbon ions with PLK1 inhibitors could potentially exploit this apparent mitotic vulnerability. PLK1 inhibition has been shown to radiosensitize p53 wild-type NSCLC cells including A549 (64, 65), though whether the transcriptional suppression we observe translates to protein-level depletion remains to be confirmed.

The selective enrichment of TNF-NF-κB signaling following carbon ion exposure raises the possibility that carbon ions may prime the tumor microenvironment for immune engagement. This could have implications for combining particle therapy with immune checkpoint inhibitors (66, 67), particularly in tumor settings where NF-κB-driven inflammatory signaling contributes to immunogenicity (68).

Conversely, X-ray exposure was associated with histone transcript upregulation at 4 h, potentially reflecting a chromatin restoration phase during which epigenetic vulnerabilities may arise, thereby suggesting a possible window for combining low-LET radiation with chromatin-targeting agents such as HDAC or BET inhibitors (69).

Finally, the differential engagement of these programs between radiation modalities, together with their potential sensitivity to p53 and KRAS mutational status, suggests that transcriptomic profiling of tumor biopsies could, in principle, help identify cellular contexts most likely to benefit from high- versus low-LET radiation, or from specific radiation-drug combinations.

These proposed transcriptional implications remain speculative propositions requiring validation in additional model systems and clinically annotated tumor material. Nevertheless, they illustrate how transcriptomic profiling may contribute to rational combination therapy design in the context of particle therapy.

### 4.5 Limitations and future directions

The transcriptional responses elicited by equal physical doses of X-rays and carbon ions were compared to investigate the transcriptional basis of the RBE difference, specifically to identify transcriptional programs that may underlie or contribute to the greater biological effectiveness of carbon ions in cell killing. The transcriptional differences observed at equal energy dose represent candidate molecular events that may contribute mechanistically to the differential biological outcomes previously documented in this model (17–19). Correcting for RBE upfront and comparing at iso-effective doses would equalize the biological outcome by design, eliminating the transcriptional signal under investigation. Therefore, a comparison of the transcriptional profile in response to iso-effective doses of X-rays and C-ions, e.g. for survival, was not performed in this work. However, based on the inherent physical characteristics of X-rays as sparsely ionizing radiation and carbon ions as densely ionizing radiation depositing energy in tracks, such a comparison could further elucidate the role of the number and the spatial distribution of ionizations inducing DNA damage (simple vs. complex damage) in eliciting the observed transcriptional response.

Transcriptomic profiling was performed at 4 h post-irradiation, reflecting a deliberate choice based on the kinetics of radiation-induced transcriptional responses. ATM/ATR activation and p53 stabilization occur within minutes of irradiation. Peak transcriptional induction of canonical DDR targets is well established within the 2–6 h interval, making 4 h appropriate for capturing the primary radiation quality-specific transcriptional wave before secondary adaptive responses, p53 autoregulatory feedback, checkpoint resolution, and radiation-induced cell death begin to confound the signal. Histone mRNA regulation and SLBP-dependent stability are likewise most clearly captured at early post-irradiation time points before replication fork dynamics resolve. Whether the transcriptional differences identified here represent transient early adaptations or persist as sustained features associated with the prolonged G2/M arrest and reduced clonogenic survival previously observed in this model (17–19) cannot be determined from the present data. Time-course transcriptomic profiling therefore represents an important priority for future work.

All experiments were performed in a single NSCLC cell line, A549, which carries a constitutively activating KRAS G12S mutation and is p53 wild-type. The transcriptional programs identified here may therefore reflect the specific genetic background of A549 cells rather than, or in addition to, general features of the high-LET radiation response. The KRAS-associated pathway enrichment, the degree of NF-κB-associated signaling, and the magnitude of checkpoint enforcement may all be influenced by constitutive RAS-MAPK activity and intact p53 signaling in this line. We note that the p53-null H358 NSCLC cell line has been characterized in our prior work under the same irradiation conditions (19), providing some contextual comparison, though without matched transcriptomics. Validation of key findings in additional NSCLC cell lines with differing p53 and KRAS status is necessary to assess the generalizability of the transcriptional programs described here and represents an important future direction.

The conclusions of this study are based on RNA-seq-derived transcript abundance and pathway inference. No independent experimental validation has been performed within the scope of this study. Key findings including PLK1 and BUB1 repression at the protein level, histone H2B and H4 protein dynamics, NF-κB nuclear translocation, and SLBP expression and phosphorylation status, remain to be confirmed by orthogonal approaches. Several functional phenotypes implied by the transcriptional data, including prolonged G2/M arrest and NF-κB pathway activity have been independently characterized in this model system in prior publications (17–19). These provide supportive context for the biological relevance of the transcriptional programs identified here. The present study should therefore be understood as a transcriptomic characterization that generates testable hypotheses for future functional validation.

The proposed model linking sustained replication fork arrest to SLBP-dependent histone mRNA destabilization is mechanistically plausible and internally consistent with the transcriptional data, but remains speculative. Direct validation would require SLBP protein quantification, histone transcript half-life measurement by metabolic labelling or actinomycin D pulse-chase, replication fork activity assessment by DNA fiber assay or EdU incorporation, and nascent transcription profiling by SLAM-seq or TT-seq. Demonstration of downstream chromatin structural changes would additionally require chromatin accessibility profiling (ATAC-seq), histone occupancy measurements (ChIP-seq), or nucleosome dynamics assays. These experiments represent important priorities for future work.

## 5 Conclusion

This study characterizes the early transcriptional response at 4 h post-irradiation to equal physical doses of high-LET carbon ions and low-LET X-rays in A549 lung cancer cells. While both radiation modalities activated a conserved p53-mediated DNA damage response, carbon ion irradiation was additionally associated with deeper repression of the replication-segregation machinery, discordant suppression of histone gene expression relative to X-rays, and selective enrichment of stress-associated inflammatory and extracellular matrix remodeling programs. Together, these features suggest a distinct transcriptional profile not observed to the same extent following X-ray exposure at equivalent physical dose.

These findings identify the replication-chromatin axis as a candidate mechanistic axis underlying carbon ion response and provide a basis for future investigation into molecular programs contributing to the enhanced biological effectiveness of carbon ion therapy.

## Data Availability

The datasets presented in this study can be found in online repositories. The names of the repository/repositories and accession number(s) can be found below: https://www.ncbi.nlm.nih.gov/geo/ GSE334631 https://www.ncbi.nlm.nih.gov/geo/, GSE334632.

## References

[1] Luo G Zhang Y Rumgay H Morgan E Langselius O Vignat J . Estimated worldwide variation and trends in incidence of lung cancer by histological subtype in 2022 and over time: a population-based study. Lancet Respir Med. (2025) 13:348–63. doi: 10.1016/S2213-2600(24)00428-4 39914442

[2] Tachihara M Tsujino K Ishihara T Hayashi H Sato Y Kurata T . Durvalumab plus concurrent radiotherapy for treatment of locally advanced non–small cell lung cancer: The DOLPHIN phase 2 nonrandomized controlled trial. JAMA Oncol. (2023) 9:1505–13. doi: 10.1001/jamaoncol.2023.3309 37676681 PMC10485744

[3] Brada M Forbes H Ashley S Fenwick J . Improving outcomes in NSCLC: Optimum dose fractionation in radical radiotherapy matters. J Thorac Oncol. (2022) 17:532–43. doi: 10.1016/j.jtho.2022.01.006 35092841

[4] Vera P Thureau S Tinier F Chaumet-Riffaud P Hapdey S Kolesnikov-Gauthier H . Adaptive radiotherapy (up to 74 Gy) or standard radiotherapy (66 Gy) for patients with stage III non-small-cell lung cancer, according to [18F]FDG-PET tumour residual uptake at 42 Gy (RTEP7–IFCT-1402): a multicentre, randomised, controlled phase 2 trial. Lancet Oncol. (2024) 25:1176–87. doi: 10.1016/S1470-2045(24)00320-6 39134086

[5] Soto-Cambres A Farré N . Is SBRT the optimal first-line treatment for operable early-stage NSCLC in elderly patients? Clin Transl Oncol. (2025) 27:3570–9. doi: 10.1007/s12094-025-03914-0 40221951

[6] Shirai K Aoki S Endo M Takahashi Y Fukuda Y Akahane K . Recent developments in the field of radiotherapy for the management of lung cancer. Jpn J Radiol. (2025) 43:186–99. doi: 10.1007/s11604-024-01663-8 39316285 PMC11790782

[7] Hada M Georgakilas A . Formation of clustered DNA damage after high-LET irradiation: A review. J Radiat Res. (2008) 49:203–10. doi: 10.1269/jrr.07123 18413977

[8] Helm A Fournier C . High-LET charged particles: radiobiology and application for new approaches in radiotherapy. Strahlenther Onkol. (2023) 199:1225–41. doi: 10.1007/s00066-023-02158-7 37872399 PMC10674019

[9] Nikitaki Z Velalopoulou A Zanni V Tremi I Havaki S Kokkoris M . Key biological mechanisms involved in high-LET radiation therapies with a focus on DNA damage and repair. Expert Rev Mol Med. (2022) 24:e15. doi: 10.1017/erm.2022.6 35357290

[10] Kumar K Kumar S Datta K Fornace AJ Suman S . High-LET-radiation-induced persistent DNA damage response signaling and gastrointestinal cancer development. Curr Oncol. (2023) 30:5497–514. doi: 10.3390/curroncol30060416 37366899 PMC10297158

[11] Kolnohuz A Ebrahimpour L Yolchuyeva S Manem VSK . Gene expression signature predicts radiation sensitivity in cell lines using the integral of dose–response curve. BMC Cancer. (2024) 24:2. doi: 10.1186/s12885-023-11634-3 38166789 PMC10763485

[12] Sagkrioti E Biz G Takan I Asfa S Nikitaki Z Zanni V . Radiation type- and dose-specific transcriptional responses across healthy and diseased mammalian tissues. Antioxidants. (2022) 11:2286. doi: 10.3390/antiox11112286 36421472 PMC9687520

[13] Amundson S . The transcriptomic revolution and radiation biology. Int J Radiat Biol. (2022) 98:428–38. doi: 10.1080/09553002.2021.1987562 34586968 PMC9520858

[14] Boo S Kim Y . The emerging role of RNA modifications in the regulation of mRNA stability. Exp Mol Med. (2020) 52:400–8. doi: 10.1038/s12276-020-0407-z 32210357 PMC7156397

[15] Wickramasinghe V Venkitaraman A . RNA processing and genome stability: Cause and consequence. Mol Cell. (2016) 61:496–505. doi: 10.1016/j.molcel.2016.02.001 26895423 PMC5905668

[16] Naro C Bielli P Pagliarini V Sette C . The interplay between DNA damage response and RNA processing: the unexpected role of splicing factors as gatekeepers of genome stability. Front Genet. (2015) 6. doi: 10.3389/fgene.2015.00142 25926848 PMC4397863

[17] Nisar H Labonté F Roggan M Schmitz C Chevalier F Konda B . Hypoxia modulates radiosensitivity and response to different radiation qualities in A549 non-small cell lung cancer (NSCLC) cells. Int J Mol Sci. (2024) 25:1010. doi: 10.3390/ijms25021010 38256084 PMC10816011

[18] Nisar H Sanchidrián González PM Labonté FM Schmitz C Roggan MD Kronenberg J . NF-κB in the radiation response of A549 non-small cell lung cancer cells to X-rays and carbon ions under hypoxia. Int J Mol Sci. (2024) 25:4495. doi: 10.3390/ijms25084495 38674080 PMC11050661

[19] Nisar H Brauny M Labonté FM Schmitz C Konda B Hellweg CE . DNA damage and inflammatory response of p53 null H358 non-small cell lung cancer cells to X-ray exposure under chronic hypoxia. Int J Mol Sci. (2024) 25:12590. doi: 10.3390/ijms252312590 39684302 PMC11641747

[20] Tinganelli W Durante M . Carbon ion radiobiology. Cancers. (2020) 12:3022. doi: 10.3390/cancers12103022 33080914 PMC7603235

[21] Salah A Wollschläger D Callari M Schmidberger H Marini F Zahnreich S . Genome-wide transcriptomic response of whole blood to radiation. Sci Rep. (2025) 15:19840. doi: 10.1038/s41598-025-04898-1 40473848 PMC12141496

[22] Gao Y Duan Q Wu N Xu B . A heterogeneous cellular response to ionizing radiation revealed by single cell transcriptome sequencing. Am J Cancer Res. (2021) 11:513–29. PMC7868766 33575084

[23] Choudhary S Burns S Mirsafian H Li W Vo DT Qiao M . Genomic analyses of early responses to radiation in glioblastoma reveal new alterations at transcription, splicing, and translation levels. Sci Rep. (2020) 10:8979. doi: 10.1038/s41598-020-65638-1 32488114 PMC7265345

[24] Nisar H Konda B Hoffmann M Labonté FM Arif M Diegeler S . Effect of reoxygenation on radioresistance of chronically hypoxic A549 non-small cell lung cancer (NSCLC) cells following X-ray and carbon ion exposure. Int J Mol Sci. (2025) 26:9153. doi: 10.3390/ijms26189153 41009714 PMC12470565

[25] Nisar H Sanchidrián González PM Brauny M Labonté FM Schmitz C Roggan MD . Hypoxia changes energy metabolism and growth rate in non-small cell lung cancer cells. Cancers Bsl. (2023) 15:2472. doi: 10.3390/cancers15092472 37173939 PMC10177580

[26] Foster K Oster C Mayer M Avery ML Audus KL . Characterization of the A549 cell line as a type II pulmonary epithelial cell model for drug metabolism. Exp Cell Res. (1998) 243:359–66. doi: 10.1006/excr.1998.4172 9743595

[27] R Core Team . R: A Language and Environment for Statistical Computing. Vienna, Austria: R Foundation for Statistical Computing (2025).

[28] Al-Arafat T Mao A Katsube T Wang B . Exploring the role of p53 in radiosensitivity: A key player in cancer therapy. Radiation. (2024) 4:309–24. doi: 10.3390/radiation4040023 30654563

[29] Okazaki R . Role of p53 in regulating radiation responses. Life. (2022) 12:1099. doi: 10.3390/life12071099 35888186 PMC9319710

[30] Jin S Mazzacurati L Zhu X Tong T Song Y Shujuan S . Gadd45a contributes to p53 stabilization in response to DNA damage. Oncogene. (2003) 22:8536–40. doi: 10.1038/sj.onc.1206907 14627995

[31] Kawase T Ohki R Shibata T Tsutsumi S Kamimura N Inazawa J . PH domain-only protein PHLDA3 is a p53-regulated repressor of Akt. Cell. (2009) 136:535–50. doi: 10.1016/j.cell.2008.12.002 19203586

[32] Kawase T Ichikawa H Ohta T Nozaki N Tashiro F Ohki R . p53 target gene AEN is a nuclear exonuclease required for p53-dependent apoptosis. Oncogene. (2008) 27:3797–810. doi: 10.1038/onc.2008.32 18264133

[33] Zhang C Liu J Wu R Liang Y Lin M Liu J . Tumor suppressor p53 negatively regulates glycolysis stimulated by hypoxia through its target RRAD. Oncotarget. (2014) 5:5535–46. doi: 10.18632/oncotarget.2137 25114038 PMC4170611

[34] Funauchi Y Tanikawa C Yi Lo PHY Mori J Daigo Y Takano A . Regulation of iron homeostasis by the p53-ISCU pathway. Sci Rep. (2015) 5:16497. doi: 10.1038/srep16497 26560363 PMC4642350

[35] Ruan X Zhang R Li R Zhu H Wang Z Wang C . The research progress in physiological and pathological functions of TRAF4. Front Oncol. (2022) 12:842072. doi: 10.3389/fonc.2022.842072 35242717 PMC8885719

[36] Di J Huang H Wang Y Qu D Tang J Cheng Q . p53 target gene Rap2B regulates the cytoskeleton and inhibits cell spreading. J Cancer Res Clin Oncol. (2015) 141:1791–8. doi: 10.1007/s00432-015-1948-8 25762091 PMC11823724

[37] Farooqi A Siddik Z . Platelet-derived growth factor (PDGF) signalling in cancer: rapidly emerging signalling landscape. Cell Biochem Funct. (2015) 33:257–65. doi: 10.1002/cbf.3120 26153649 PMC5289298

[38] Junqueira Alves C Dariolli R Haydak J Kang S Hannah T Wiener RJ . Plexin-B2 orchestrates collective stem cell dynamics via actomyosin contractility, cytoskeletal tension and adhesion. Nat Commun. (2021) 12:6019. doi: 10.1038/s41467-021-26296-7 34650052 PMC8517024

[39] Pecorari R Bernassola F Melino G Candi E . Distinct interactors define the p63 transcriptional signature in epithelial development or cancer. Biochem J. (2022) 479:1375–92. doi: 10.1042/BCJ20210737 35748701 PMC9250260

[40] Kudo K Tsuyama N Nagata K Imaoka T Iizuka D Sugai-Takahashi M . ΔNp63α transcriptionally represses p53 target genes involved in the radiation-induced DNA damage response. Radiat Oncol. (2022) 17:183. doi: 10.1186/s13014-022-02139-7 36380314 PMC9667649

[41] Gómez R Viera A Moreno-Mármol T Berenguer I Guajardo-Grence A Tóth A . Kinase PLK1 regulates the disassembly of the lateral elements and the assembly of the inner centromere during the diakinesis/metaphase I transition in male mouse meiosis. Front Cell Dev Biol. (2023) 10. doi: 10.3389/fcell.2022.1069946 36733339 PMC9887526

[42] Kreis N-N Moon HH Wordeman L Louwen F Solbach C Yuan J . KIF2C/MCAK a prognostic biomarker and its oncogenic potential in Malignant progression, and prognosis of cancer patients: a systematic review and meta-analysis as biomarker. Crit Rev Clin Lab Sci. (2024) 61:404–34. doi: 10.1080/10408363.2024.2309933 38344808 PMC11815995

[43] Chen Q Zhang M Pan X Yuan X Zhou L Yan L . Bub1 and CENP-U redundantly recruit Plk1 to stabilize kinetochore-microtubule attachments and ensure accurate chromosome segregation. Cell Rep. (2021) 36:109740. doi: 10.1016/j.celrep.2021.109740 34551298

[44] Liu C Chen Y Deng Y Dong Y Jiang J Chen S . Survival-based bioinformatics analysis to identify hub genes and key pathways in non-small cell lung cancer. Trans Cancer Res. (2019) 8(4):1188–98. doi: 10.21037/tcr.2019.06.35 35116861 PMC8797769

[45] Rajpurohit Y Sharma D Lal M Soni I . A perspective on tumor radiation resistance following high-LET radiation treatment. J Cancer Res Clin Oncol. (2024) 150:226. doi: 10.1007/s00432-024-05757-8 38696003 PMC11065934

[46] Chen Z Wang X Gao X Arslanovic N Chen K Tyler JK . Transcriptional inhibition after irradiation occurs preferentially at highly expressed genes in a manner dependent on cell cycle progression. eLife. (2024) 13:RP94001. doi: 10.7554/eLife.94001 39392398 PMC11469672

[47] Su C Gao G Schneider S Helt C Weiss C O'Reilly MA . DNA damage induces downregulation of histone gene expression through the G1 checkpoint pathway. EMBO J. (2004) 23:1133–43. doi: 10.1038/sj.emboj.7600120 14976556 PMC380976

[48] Pan C Fan Y . Role of H1 linker histones in mammalian development and stem cell differentiation. Biochim Biophys Acta. (2016) 1859:496–509. doi: 10.1016/j.bbagrm.2015.12.002 26689747 PMC4775330

[49] Duronio R Marzluff W . Coordinating cell cycle-regulated histone gene expression through assembly and function of the histone locus body. RNA Biol. (2017) 14:726–38. doi: 10.1080/15476286.2016.1265198 28059623 PMC5519241

[50] Zou K Zeng Z . Role of early growth response 1 in inflammation-associated lung diseases. Am J Physiol Lung Cell Mol Physiol. (2023) 325:L143–54. doi: 10.1152/ajplung.00413.2022 37401387 PMC10511164

[51] Liu S Li Z Lan S Hao H Baz AA Yan X . The dual roles of activating transcription factor 3 (ATF3) in inflammation, apoptosis, ferroptosis, and pathogen infection responses. Int J Mol Sci. (2024) 25:824. doi: 10.3390/ijms25020824 38255898 PMC10815024

[52] Nishad S Ghosh A . Gene expression of immediate early genes of AP-1 transcription factor in human peripheral blood mononuclear cells in response to ionizing radiation. Radiat Environ Biophys. (2016) 55:431–40. doi: 10.1007/s00411-016-0662-5 27586508

[53] Beach C MacLean D Majorova D Arnold JN Olcina MM . The effects of radiation therapy on the macrophage response in cancer. Front Oncol. (2022) 12:1020606. doi: 10.3389/fonc.2022.1020606 36249052 PMC9559862

[54] Janus P Szołtysek K Zając G Stokowy T Walaszczyk A Widłak W . Pro-inflammatory cytokine and high doses of ionizing radiation have similar effects on the expression of NF-kappaB-dependent genes. Cell Signal. (2018) 46:23–31. doi: 10.1016/j.cellsig.2018.02.011 29476964

[55] Mathew B Takekoshi D Sammani S Epshtein Y Sharma R Smith BD . Role of GADD45a in murine models of radiation- and bleomycin-induced lung injury. Am J Physiol Lung Cell Mol Physiol. (2015) 309:L1420–9. doi: 10.1152/ajplung.00146.2014 26498248 PMC4683317

[56] Betzler AC Theodoraki M-N Schuler PJ Döscher J Laban S Hoffmann TK . NF-κB and its role in checkpoint control. Int J Mol Sci. (2020) 21:3949. doi: 10.3390/ijms21113949 32486375 PMC7312739

[57] Allgayer H Mahapatra S Mishra B Swain B Saha S Khanra S . Epithelial-to-mesenchymal transition (EMT) and cancer metastasis: the status quo of methods and experimental models 2025. Mol Cancer. (2025) 24:167. doi: 10.1186/s12943-025-02338-2 40483504 PMC12144846

[58] Guerrero Quiles C Fahy S Bartak M Gonzalez Abalos J Powell E Lodhi T . Radiation-induced extracellular matrix remodelling drives prognosis and predicts radiotherapy response in muscle-invasive bladder cancer. Front Oncol. (2025) 15:1616943. doi: 10.3389/fonc.2025.1616943 40792276 PMC12336036

[59] Beck M Moreels M Quintens R Abou-El-Ardat K El-Saghire H Tabury K . Chronic exposure to simulated space conditions predominantly affects cytoskeleton remodeling and oxidative stress response in mouse fetal fibroblasts. Int J Mol Med. (2014) 34:606–15. doi: 10.3892/ijmm.2014.1785 24859186

[60] Pereira F Ferreira A Reis CA Sousa MJ Oliveira MJ Preto A . KRAS as a modulator of the inflammatory tumor microenvironment: therapeutic implications. Cells. (2022) 11:398. doi: 10.3390/cells11030398 35159208 PMC8833974

[61] Sabio G Davis RJ . TNF and MAP kinase signaling pathways. Semin Immunol. (2014) 26:237–45. doi: 10.1016/j.smim.2014.02.009 24647229 PMC4099309

[62] Pokharel SM Chiok K Shil NK Mohanty I Bose S . Tumor necrosis factor-alpha utilizes MAPK/NFκB pathways to induce cholesterol-25 hydroxylase for amplifying pro-inflammatory response via 25-hydroxycholesterol-integrin-FAK pathway. PloS One. (2021) 16:e0257576. doi: 10.1371/journal.pone.0257576 34551004 PMC8457477

[63] Mitchell JL Mutch NJ . Let’s cross‐link: diverse functions of the promiscuous cellular transglutaminase factor XIII‐A. J Thromb Haemostasis. (2019) 17:19–30. doi: 10.1111/jth.14348 30489000

[64] Inoue M Yoshimura M Kobayashi M Morinibu A Itasaka S Hiraoka M . PLK1 blockade enhances therapeutic effects of radiation by inducing cell cycle arrest at the mitotic phase. Sci Rep. (2015) 5:15666. doi: 10.1038/srep15666 26503893 PMC4621528

[65] Van den Bossche J Domen A Peeters M Deben C De Pauw I Jacobs J . Radiosensitization of non-small cell lung cancer cells by the Plk1 inhibitor volasertib is dependent on the p53 status. Cancers. (2019) 11:1893. doi: 10.3390/cancers11121893 31795121 PMC6966428

[66] Takahashi Y Yasui T Minami K Tamari K Hayashi K Otani K . Carbon ion irradiation enhances the antitumor efficacy of dual immune checkpoint blockade therapy both for local and distant sites in murine osteosarcoma. Oncotarget. (2019) 10:633–46. doi: 10.18632/oncotarget.26551 30774761 PMC6363009

[67] Helm A Totis C Durante M Fournier C . Are charged particles a good match for combination with immunotherapy? Current knowledge and perspectives. Int Rev Cell Mol Biol. (2023) 376:1–36. doi: 10.1016/bs.ircmb.2023.01.001 36997266

[68] Simon PS Bardhan K Chen MR Paschall AV Lu C Bollag RJ . NF-κB functions as a molecular link between tumor cells and Th1/Tc1 T cells in the tumor microenvironment to exert radiation-mediated tumor suppression. Oncotarget. (2016) 7:23395–415. doi: 10.18632/oncotarget.8246 27014915 PMC5029635

[69] Wang P Yuan D Guo F Chen X Zhu L Zhang H . Chromatin remodeling modulates radiosensitivity of the daughter cells derived from cell population exposed to low- and high-LET irradiation. Oncotarget. (2017) 8:52823–36. doi: 10.18632/oncotarget.17275 28881774 PMC5581073

