# Supplementary material for: Transcriptomic differences in chromatin and cell cycle regulation in A549 cells after irradiation with carbon ions and X-rays
Source: Front Oncol. 2026 Jul 14;16:1869974. doi: 10.3389/fonc.2026.1869974 (PMC13408517; doi:10.3389/fonc.2026.1869974)
Supplement: Supplementary file 1 [file DataSheet1.docx]

**Supplementary Figures**

| 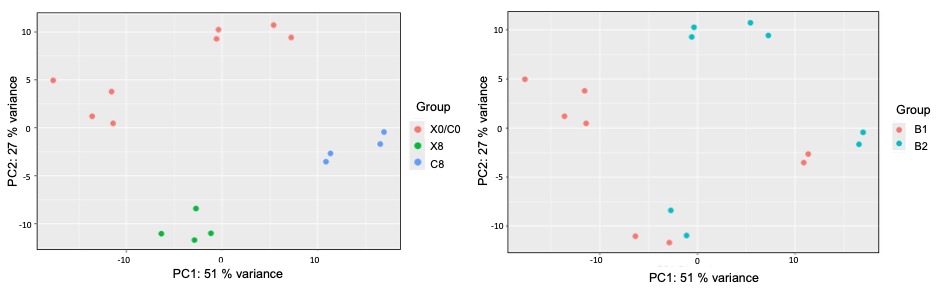 |
| --- |
| **Figure A1. Principal component analysis (PCA) of variance-stabilized count data.**  PCA was performed on variance-stabilized transformed (VST) counts using the top 500 most variable genes. Each point represents one sample.  X0/C0: Unirradiated cells (n=8);  X8: X-ray irradiated cells, 8 Gy (n=4);  C8: Carbon ion irradiated cells, 8 Gy (n=4).  PC1 (51% variance) separates irradiated from unirradiated samples and further distinguishes carbon ion from X-ray conditions. PC2 (27% variance) captures residual biological variability between replicates.  Left panel: samples colored by experimental condition.  Right panel: same PCA colored by irradiation batch (B1, B2). Samples from both batches are interleaved with no systematic batch-driven clustering, confirming that batch is not a dominant source of transcriptional variation and that batch correction does not confound the differential expression analysis. |

| 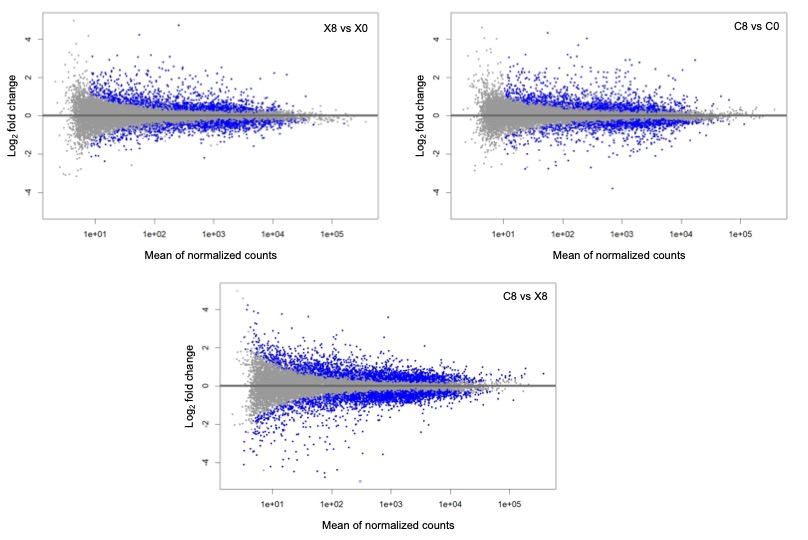 |
| --- |
| **Figure A2.** MA plots for all pairwise irradiation contrasts. Each point represents one gene. Log2 fold change (LFC) is plotted against mean normalized counts. Significant genes (adjusted p-value < 0.05) are shown in blue. The horizontal line indicates LFC = 0. Genes with LFC values outside the displayed range (±5) are represented as triangles at the plot boundary.  Top left panel: X-ray irradiation versus unirradiated control (X8 vs. X0).  Top right panel: Carbon ion irradiation versus unirradiated control (C8 vs. C0).  Bottom panel: Carbon ion versus X-ray irradiation (C8 vs. X8). |
